# A Comprehensive Review of Molecular Mechanisms, Pharmacokinetics, Toxicology and Plant Sources of Juglanin: Current Landscape and Future Perspectives

**DOI:** 10.3390/ijms251910323

**Published:** 2024-09-25

**Authors:** Magdalena Rutkowska, Martyna Witek, Monika A. Olszewska

**Affiliations:** Department of Pharmacognosy, Faculty of Pharmacy, Medical University of Lodz, 1 Muszyńskiego St., 90-151 Lodz, Poland; maniawit00@gmail.com (M.W.); monika.olszewska@umed.lodz.pl (M.A.O.)

**Keywords:** juglanin, molecular mechanisms, toxicology, pharmacokinetics, plant materials, future perspectives

## Abstract

Juglanin (kaempferol 3-*O*-α-L-arabinofuranoside) is a flavonol glycoside occurring in many plants, including its commercial sources *Juglans regia*, *Polygonum aviculare* and *Selliguea hastata*. Recent extensive studies have explored the potential of using juglanin in various pathological conditions, including cardiovascular disorders, central nervous and skeletal system disorders, metabolic syndrome, hepatic injury, and cancers. The results indicated a wide range of effects, like anti-inflammatory, anti-oxidant, anti-fibrotic, anti-thrombotic, anti-angiogenic, hepatoprotective, hypolipidemic, hypoglycemic, anti-apoptotic (normal cells), and pro-apoptotic (cancer cells). The health-promoting properties of juglanin can be attributed to its influence on many signaling pathways, associated with SIRT1, AMPK, Nrf2, STING, TLR4, MAPKs, NF-κB, AKT, JAK, and their downstream genes. This review primarily summarizes the current knowledge of molecular mechanisms, pharmacokinetics, biocompatibility, and human use safety of juglanin. In addition, the most promising new plant sources and other existing challenges and prospects have also been reviewed and discussed, aiming to provide direction and rationale for the further development and broader pharmaceutical application of juglanin.

## 1. Introduction

For centuries, plants have served as not just food or building materials but also as a rich base of therapeutic benefits. Today, they continue to be a subject of extensive research as potential sources of molecules for drug development. Among the critical components of plant products and leading model natural drugs are flavonoids, a vast family with over 8000 known representatives in the plant kingdom. While industrial compounds and clinically approved medicines like rutin, diosmin, hesperidin, or silybin have emerged from this group, the potential of flavonoids is still being intensely evaluated, with numerous other members showing promising biological properties [1].

**Figure 1:**
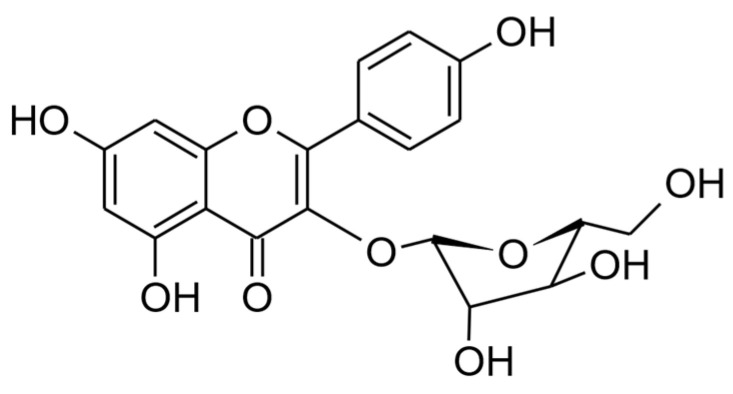
The chemical structure of juglanin.

Juglanin (kaempferol 3-*O*-α-L-arabinofuranoside, CAS 5041-67-8, Figure 1) is a member of a large group of flavonols and a compound that has garnered significant attention in recent years. It is found in a variety of plant species, including *Juglans* spp., e.g., *Juglans regia* L. (hence its common name), *Polygonum aviculare* L., *Selliguea hastata* (Thunb.) H. Ohashi and K. Ohashi (these last three being commercial sources of juglanin), *Rubus* spp., *Prunus* spp., *Rosa* spp., etc. [2,3,4,5]. Alongside astragalin, tiliroside, and kaempferol 3-*O*-rutinoside, juglanin is one of the most prevalent natural flavonols within the kaempferol glycoside group, although this group is less widespread in the plant kingdom than quercetin glycosides, such as rutin or isoquercitrin.

The exploration of juglanin’s biological activity has a rich history, dating back to 1984. However, the bulk of research has been conducted in 2020–2023, indicating a significant surge in interest. These studies, which encompass mainly in vivo animal models and in vitro cellular studies, have explored the potential of using juglanin for a wide range of conditions. The anti-inflammatory, anti-oxidative, anti-fibrotic, and anti-apoptotic (or pro-apoptotic in the case of cancer cells) properties of juglanin have been identified as its primary modes of action. The signaling pathways targeted by the compound, including those dependent on the 5′AMP-activated protein kinase (AMPK), mitogen-activated protein kinases (MAPKs), nuclear factor kappa-light-chain-enhancer of activated B cells (NF-kB), and many others, have been extensively studied, further validating its potential [6,7,8,9,10,11,12,13].

The wealth of accumulated results, pointing to the unique and promising features of juglanin, forms the foundation of this paper. The review primarily focuses on the molecular mechanisms of juglanin activity but also delves into its pharmacokinetic properties, biocompatibility, and human use safety. In addition, the known plant sources of juglanin were reviewed, with a particular emphasis on the existing and most promising industrial plant materials for its acquisition. The paper summarizes the significant scientific progress in understanding the biological properties of juglanin in recent years and discusses the current challenges and prospects of using juglanin in therapy, aiming to provide direction and rationale for further development and broader application of this undoubtedly exciting molecule.

## 2. Juglanin Occurrence in Plant Species

Juglanin has been detected in different plant species from divergent botanical families, such as Anacardiaceae R. Br., Annonaceae Juss., Cornaceae Bercht. ex J. Presl, Crassulaceae J. St.-Hil., *Cupressaceae* Gray, Dryopteridaceae Herter, Ericaceae Juss., Euphorbiaceae Juss., Fabaceae Juss., Geraniaceae Juss., Juglandaceae DC. ex Perleb, Lauraceae Juss., Lamiaceae Martinov, Myrtaceae Juss., Onagraceae Juss., Polypodiaceae J. Presl & C. Presl, Polygonaceae Juss., Rosaceae Juss., and Sapindaceae Juss.

The plant parts in which juglanin has been observed are flowers, leaves, herbs, fruits, and seeds. Table 1 presents the whole list of species where juglanin was detected (in alphabetical order), plant parts and type of extracts used in the studies, juglanin content (if determined in the study), and methods used for analysis.

Commercially available juglanin is obtained from *Juglans regia* L. (probably leaves, although the detailed information is proprietary to the manufacturer), as well as herbs of *Polygonum aviculare* L. (European Pharmacopoeia material) and *Selliguea hastata* (Thunb.) H.Ohashi and K.Ohashi (a species of traditional Chinese medicine). From these, *Juglans regia* seems to be the most reasonable choice because of its wide cultivation and high productivity of leaves, which are waste products of walnut acquisition and seed oil production. Although the leaves are also traditional herbal medicines, their medicinal application is limited by the presence of toxic juglone [59]. On the other hand, juglone is a powerful allelochemical, extensively tested for agricultural use as a natural insecticide, bactericide, and fungicide [60]. Therefore, the simultaneous isolation of juglanin and juglone is valuable for both agriculture and pharmaceutical industries, making the process more economically viable.

The available literature about juglanin content in plants is limited (Table 1); however, for the majority of species (including the commercial sources), it indicates relatively low levels calculated per dry weight (dw) of plant materials (0.2–1.6 mg/g dw) or extracts (1.0–3.6 mg/g dw). Moreover, numerous accompanying compounds [26,39] often make the isolation procedures difficult and expensive; consequently, the price of juglanin is about 350 euros for 5 mg. For some plant materials, the reported content of juglanin was higher, i.e., for *Prunus serotina* Ehrh. leaves (about 2.0–2.2 mg/g dw), *Prunus spinosa* L. flowers (2.6–5.7 mg/g dw), and ripe *Rubus crataegifolius* Bunge fruits (5.0 mg/g dw) [5,41,46]. This data translates into various isolation yields of juglanin from different species, ranging from 1–8 mg per 1 kg of respective plant material of *Afgekia mahidoliae* B. L. Burtt and Chermsir., *Geranium platyanthum* Duthie, *Ludwigia adscendens* (L.) H. Hara, *Mallotus nanus* Airy Shaw, *Persea americana* Mill., *Polygonum aviculare* L., *Selliguea hastata* (Thunb.) H. Ohashi and K. Ohashi, *Rubus rigidus* Sm., *Rosa chinensis* Jacq., *Rosa damascene* Herrm., or *Prunus serrulata* Lindl. [3,15,25,30,31,36,43,48,49,52,56], through 40–58 mg/kg separated from *Xylopia parviflora* Spruce fruits, *Myrcia tomentosa* (Aubl.) DC. leaves, or *Prunus spinosa* L. leaves [33,44,58], up to 1140–1160 mg/kg achieved form *Prunus serotina* Ehrh. leaves or *Prunus spinosa* L. flowers [42,61]. However, the reported yields come from laboratory-scale experiments, focused mainly on determining the chemical structure of isolates or their activity testing, without systematic optimization of isolation procedures. Therefore, they may not reflect the real productivity of the plant materials in terms of juglanin isolation, especially in an industrial context. Nevertheless, searching for new, efficient sources of the compound and developing performant methods of its isolation is necessary for the broader use of this compound. At present, the estimated cost of the juglanin needed to carry out the 4-week animal study on ten rats per group using the lowest effective dose (taking into account the bioavailability and activity studies described later in the manuscript and the cost of juglanin available on the market) is about 80,000 euros. Therefore, considering the potential doses needed for use in humans, it makes the clinical application of juglanin many times more expensive.

Currently, two *Prunus* species (i.e., *Prunus serotina* Ehrh. leaves and *Prunus spinosa* L. flowers) and *Rubus crataegifolius* Bunge fruits seem to be the most promising candidates for large-scale and cost-effective isolation of juglanin. *Prunus serotina* Ehrh., a plant native to North America but now naturalized on other continents, is known mainly in the wood industry, being used for furniture and floorboard production, or utilized as a fuel [62]. Its bark is also known in Native American ethnomedicine [63]. In this light, the leaves are a waste material that could be successfully applied for juglanin isolation. The second species, *Prunus spinosa* L., is a traditional medicinal plant and an architectural species widespread and cultivated in Europe. The *P. spinosa* flowers are valued in phytotherapy and thus easily accessible on the market as an isolation material [64]. As for *Rubus crataegifolius* Bunge, it is a wild fruit species in southeastern Asian countries, valued for its resistance to pests and environmental conditions, and thus widely used in breeding programs as a wellspring of desirable horticultural traits [65].

## 3. Pharmacokinetic Properties

### 3.1. Absorption, Distribution, Metabolism, and Excretion Based on the ADMETlab Webserver

Investigating the pharmacokinetic properties of a new chemical compound is an essential step in drug development. Understanding how the body interacts with administered substances is crucial for determining the correct dosage to provide the most significant benefit at the lowest risk of potential side effects.

The ADME of juglanin, i.e., absorption, distribution, metabolism, and excretion, were analyzed using the ADMETlab 2.0 web server [66]—an online platform for predicting the pharmacokinetics and toxicity properties of chemicals based on their structures, increasingly used in the design and search for new drugs.

The intestinal absorption of juglanin was estimated by calculating the predicted permeability level of Caco-2 (human colon adenocarcinoma line) and MDCK (Madin–Darby canine kidney) cells. While the results suggested poor Caco-2 permeability (−5.835 log cm/s, while for proper permeability it should be >−5.15), the MDCK permeability was considered as high (10^−5^ cm/s, i.e., >20 × 10^−6^ cm/s). Moreover, the probability of being the P-glycoprotein (efflux transporter) substrate was defined as medium (0.623, i.e., 0.3–0.7), meaning the moderate probability for juglanin to be effluxed back into the intestinal lumen and excreted out of the body instead of being transported to the bloodstream. Overall, the probability of human intestinal absorption at the level <30% was low (0.264), meaning a good absorption profile. Finally, the human oral bioavailability was estimated at between 20% and 30%, thus at the upper bioavailability range reported for different flavonols (2–44%) [67], which were, e.g., 2–20% for kaempferol (juglanin aglycone) and 12% for icariin (8-prenyl-kaempferol 3,7-*O*-diglucoside).

The next stage of ADME analysis was the examination of distribution parameters. The predicted value of plasma protein binding was 91.32% (optimal value for a drug should be <90%), suggesting that juglanin may have a high affinity to bind with proteins and, therefore, a low therapeutic index. On the other hand, the fraction of juglanin unbound to serum proteins was calculated within the range of 5–20% (10.7%), which can be interpreted as moderate. The volume of distribution, a theoretical concept that relates the total amount of drug in the body to the plasma concentration of the drug, was predicted as 0.988 L/kg, which can be interpreted as the proper value. The low volume of distribution (<0.04 L/kg) is observed for drugs that have a propensity to remain in the plasma, meaning a lower dose of a drug is required to achieve a given plasma concentration, but at the same time, the drug is less distributed to other tissues. The opposite is true in the case of high volume of distribution (>20 L/kg). Eventually, the probability for juglanin to cross the blood-brain barrier (BBB) was calculated as low (0.016), indicating a low chance of side effects from the central nervous system. On the other hand, based on this prediction, juglanin should not be active in the central nervous system, which is in contrast to the results of animal studies described in Section 5.6. This situation shows that computer analysis can only indicate a direction and should be confirmed by in vitro/in vivo tests.

Analysis of the interaction of juglanin with the human cytochrome P450 family suggests no inhibitory effects on CYP1A2, CYP2C19, CYP2C9, CYP2D6, CYP3A4, i.e., isoenzymes responsible for approximately 80% of the metabolism attributed to CYP450. Therefore, the potential of juglanin to impair the metabolism of other drugs seems to be low. From the tested enzymes, juglanin may be a substrate of CYP2C9 (probability at 0.792 level).

As for the excretion aspect, two parameters were calculated. The drug clearance, defined as the volume of plasma cleared of a drug over a specified time, was 4.893 mL/min/kg (clearance <5 mL/min/kg is low). Moreover, the probability of juglanin having a long half-life (>3h) was predicted as high (0.841). Therefore, eliminating juglanin from the body may take a long time, and its activity may be extended (also considering the possibility of binding to proteins). All these conclusions should be considered while determining the potential dosage for optimal activity with the lowest side effects.

### 3.2. Bioavailability Assessment Based on Experimental Studies

So far, only one in vivo study on the male Wistar rats model (n = 5) [68] has confirmed some of the juglanin pharmacokinetic properties described above, and the rest can only be approximated based on research on related compounds.

The oral administration of 600 mg/kg of *Polygonum aviculare* flavonoids from aerial parts (purified fraction of avicularin 113 mg, miricitrin 100 mg, and juglanin 41.6 mg) resulted in a concentration of juglanin in the plasma after 5 min of about 1 µg/mL. The peak concentration was after 30 min (C_max_ about 3.5 µg/mL), and after 240 min it still remained at 1 µg/mL, suggesting rapid absorption and slow elimination of juglanin. It was confirmed on the cumulative excretion test of flavonoids in urine when the maximum concentration of juglanin appeared between 15 and 24 h. However, only about 5% of the juglanin dose was eliminated unchanged, suggesting extensive metabolism (juglanin metabolites were indeed visible in the chromatographic profiles of urine samples but were not marked) [68]. This follows various studies of urinary excretion of flavonoid metabolites, where the metabolites with an intact flavonoid structure were present in urine as only 0.1–3.6% of the ingested flavonols [69].

The C_max_ of 3.5 µg/mL (i.e., 8.4 µM) of juglanin in rat plasma samples [68] seems to be at the upper limit reported for different flavonols (C_max_ 0.3–7.6 µM), as well as other flavonoids (C_max_ 0.01–6.7 µM) [69,70]. Moreover, considering the sample of plasma chromatogram after oral administration of *Polygonum aviculare* flavonoids, there were some peaks not identified, which again might be the products of flavonoid metabolism. Furthermore, the discussed work was only one study of juglanin bioavailability, and the number of animals was limited. Therefore, this aspect needs further investigation.

There are several ways in which juglanin may be transported across the intestine. First, as a glycoside it can be transported into epithelial cells by the sodium-dependent glucose transporter (SGLT). Some absorption studies of various glycosides suggested this route, but at the same time, it was found not to be the case in other examinations [71]. This may be because some flavonoid glycosides were proved to be SGLT inhibitors. However, in the case of kaempferol 3-*O*-arabinofuranoside (juglanin), kaempferol 3-*O*-arabinopyranoside, as well as kaempferol as an aglycone, no inhibition was noticed (in a study of 67 different flavonoids) [72]. Therefore, the considerable amounts of intact juglanin in the rat plasma shortly after its administration (see two paragraphs above) [68] may be because of SGLT transport (no other flavonoid occurring in the tested *Polygonum aviculare* fraction was an SGLT inhibitor). Hence, it is also essential for the absorption of flavonoids to consider a food matrix within which they are taken, and this correlation was confirmed in many previous studies [69].

The second way for juglanin to be absorbed is after its cleavage by intestinal microbiota. Arabinofuranosidases are enzymes produced by gut microbiota that may hydrolyze the L-glycosidic bond in the juglanin and to yield absorbable aglycon. Aglycones may be also further transformed by gut microbiota to phenolic acid metabolites or metabolized by, e.g., UDP-glucuronosyltransferases in the colon to glucuronides [73]. Indeed, the major metabolite identified in plasma and urine after ingesting 150 g of cooked endive (9 mg kaempferol, human study on four healthy males and four healthy females) was kaempferol-3-glucuronide. Also, free kaempferol was detected at the level of 40% of total kaempferol in the plasma [74].

Considering the above findings, the doses of juglanin selected for activity tests (discussed in Section 5), i.e., 7.5–80 mg/kg/day p.o. for animal studies, and 0.5–120 µM for cellular studies (more than half of the studies included doses ≤10 µM), appear to be reasonable. However, it is clear that pharmacokinetic properties of juglanin still hold significant potential for further research.

## 4. Safety of Use

### 4.1. The Toxicology Study Based on the ADMETlab Webserver

The potential toxicology of juglanin was analyzed with the use of the ADMETlab 2.0 webserver [66]—an online platform for the predictions of pharmacokinetic and toxicity properties of chemicals based on their structures. The probability of being hepatotoxic, carcinogenic, acting as the blocker of the hERG (human ether-a-go-go-related gene that plays a major role in the regulation of cardiac depolarization and repolarization), or causing respiratory toxicity was calculated as very low. Also, juglanin was predicted to be non-corrosive to eyes and have a medium probability (0.419 in the scale 0–1) of being an eye irritant. The rat oral toxicity was calculated as low (>500 mg/kg), and the maximum recommended daily dose (an estimate of the toxic dose threshold in humans) was defined as “excellent” (very low probability of being harmful in a dose up to 0.011 mmol/kg/day). While the Ames toxicity (a mutagenicity test based on mutation in the bacterial DNA) was calculated as highly probable, it is known that Ames-positive substances are not necessarily harmful for humans since the test may give false-positive results [75]. Finally, the high probability of skin sensitization might be a potential side effect in dermatological applications.

All in all, based on these computational studies, juglanin seems to be fairly safe to use. However, this requires an experimental confirmation in cellular in vitro and in vivo studies.

### 4.2. The Cellular Safety Studies

The cellular safety of juglanin was tested across a diverse range of cell lines, including lung, kidney, liver, endothelial, breast, microglial, astrocytes, bone marrow stromal, chondrocytes, dermal fibroblasts, and keratocytes (for details see Table 2). The methods employed to test cell viability, such as the MTT, MTS, or CCK-8 assays, were designed to measure cellular metabolic activity, specifically the capability of NAD(P)H-dependent cellular oxidoreductases to reduce different tetrazolium dyes. Moreover, for human liver cells (line L02), the release of LDH (lactate dehydrogenase) was tested to evaluate damage to the plasma membrane.

The results indicated that for the majority of cell types, juglanin was safe at a wide range of concentrations (sometimes up to 160–250 µM). Among cells that seemed to be most sensitive to the juglanin treatment were breast cells (HCC1937, HGC-27) and aortic endothelial cells, with viability levels of about 70–80% at 40 µM and 75–85% at 25–50 µM, respectively. However, these concentrations were significantly higher than those required for therapeutic activity (see Section 5, where the biological activity is discussed) and higher than observed in animal plasma after consuming flavonoids (see Section 3.2., where the bioavailability is discussed). Still, higher concentrations that can be reached locally, e.g., in the intestine, should be further tested using appropriate cell lines.

Notably, while juglanin demonstrated safety for normal breast, lung, or skin cells, it significantly disrupted the viability of corresponding cancer cells (see Section 5.8: Juglanin and anti-cancer potential) [8,77,81]. This discovery is highly promising, suggesting that juglanin could be a potential candidate for further studies as an anti-cancer compound (in addition to other activities described in the appropriate chapters), with minimal possibility of side effects on healthy cells. This potential might open up new avenues for cancer research and treatment.

### 4.3. Safety of Use Based on Animal Models

Considering the biosafety issue as being of great importance for potential clinical use of pharmaceuticals, some authors decided to verify the safety of juglanin prior to or simultaneously with biological activity studies (see Section 5) [6,8,9].

In the study of Ren et al. [6] on male C57BL/6N mice (n = 6; juglanin 30 mg/kg/day p.o. for 8 weeks), no significant difference in the histological features of major organs (i.e., liver; heart; kidney; spleen; lung), as well as hematological markers for hepatic, renal and cardiac toxicity (i.e., ALT, alanine aminotransferase; AST, aspartate transaminase; BUN, blood urea nitrogen; CRE, creatinine; CK-MB, creatine kinase isoenzymes) were observed between the control group and juglanin-treated group. Similarly, in the study of Sun et al. [9] (C57BL/6J mice, n = 8/group; juglanin 80 mg/kg/day p.o. for 30 days), there were no evident histological damages in liver, heart, kidney and spleen, and no changes in AST, ALT, BUN, CRE, and LDH. Finally, juglanin at 10–30 mg/kg/day p.o. for 28 days was non-toxic in liver, renal and lung tissues (histological analysis) of healthy nude mice (n = 6/group) [8]. In the case of potential blood-brain-barrier permeability, it is also essential that juglanin (30 mg/kg/day p.o. for 4–8 weeks) did not affect the memory and cognitive function of healthy rats or mice but, at the same time, improved them in case of sick individuals (for details see Section 5.6) [6,83].

However, it is important to note that the studies mentioned above primarily focus on active doses of juglanin. There still needs to be more evidence regarding its effects at higher doses, the potential toxicity of such doses, and the long-term effects of juglanin consumption. These gaps in knowledge highlight the need for further research in this area.

## 5. Biological Activity

This review includes 47 articles in which the biological activity of juglanin has been investigated. In Table 3, the details of all studies are presented, including the disease model, the type and parameters of the study, tested doses, the kind of activity tested, and the detailed results. Table 3 also indicates in which of the following subsections (Section 5.1, Section 5.2, Section 5.3, Section 5.4, Section 5.5, Section 5.6, Section 5.7, Section 5.8, Section 5.9, Section 5.10) it is mainly discussed (division according to diseases/body systems). The discussion part concentrates on the summary of results presented in Table 3 with special emphasis on the investigated or potential mechanisms of activity, conclusions that may be drawn from the research performed, and issues requiring further studies.

### 5.1. Juglanin and Fibrosis Treatment

**Figure 2:**
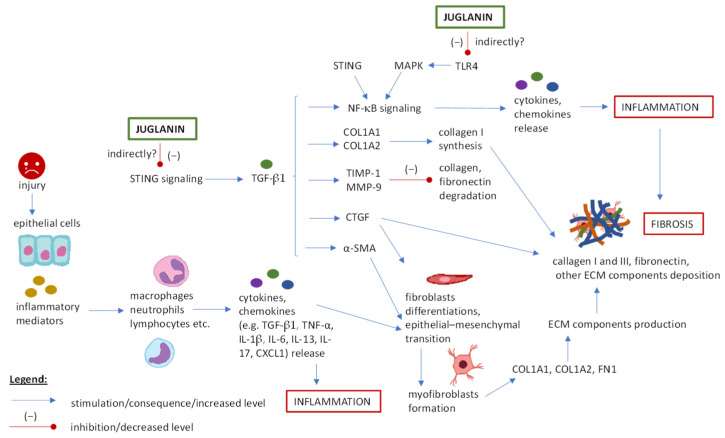
The mechanism of fibrosis with potential grip points for juglanin activity. Abbreviations: collagen type I alpha 1 (COL1A1); collagen type I alpha 2 (COL1A2); connective tissue growth factor (CTGF); chemokine C-X-C motif ligand 1 (CXCL1); extracellular matrix (ECM); fibronectin 1 (FN1); interleukin 17 (IL-17); interleukin 18 (IL-18); interleukin 1β (IL-1β); interleukin 6 (IL-6); mitogen activated protein kinase (MAPK); matrix metalloproteinase 9 (MMP-9); nuclear factor-κB (NF-κB); stimulator of interferon genes (STING); transforming growth factor β1 (TGF-β1); tissue inhibitor of metalloproteinase 1 (TIMP-1); toll-like receptor 4 (TLR4); tumor necrosis factor-α (TNF-α); α-smooth muscle actin (α-SMA).

Fibrosis, defined as pathological wound healing, is characterized by excessive build-up of extracellular matrix (ECM) components in various tissues of the body such as lungs, kidneys, liver, brain, or heart. While the formation of fibrotic tissue is a crucial part of the healing process, repeated injuries and chronic inflammation can result in progressive fibrosis, disrupt tissue structure, and lead to organ dysfunction [100]. Figure 2 displays a graph of fibrosis mechanisms, which includes key points related to juglanin activity [100,101,102,103,104] discussed below.

The anti-fibrotic potential of juglanin was tested in a mouse model of idiopathic pulmonary fibrosis (IPF) induced by bleomycin [9]. The histological changes in pulmonary tissues (percentage of fibrotic area, neutrophil infiltration, lung injury score) were significantly reduced both in simultaneous juglanin and bleomycin-treated groups and in delayed juglanin treatment (from the 10th day of bleomycin administration, late stage of fibrosis). This suggested the ability of juglanin to prevent both fibrosis development and fibrosis progression and in all experiments, the survival rate of laboratory animals was improved. In the presented study, juglanin acted like an anti-inflammatory agent by reducing levels of neutrophiles, macrophages, and pro-inflammatory cytokines (tumor necrosis factor-α, TNF-α; interleukin 6, IL-6) and chemokines (chemokine C-X-C motif ligand 1, CXCL1) in bronchoalveolar lavage fluid and lungs. Moreover, juglanin therapy whittled down the level of the crucial profibrotic cytokine, i.e., TGF-β1 (transforming growth factor beta 1), involved in the cascade reaction of trans-differentiation of fibroblasts to myofibroblasts, collagen deposition and fibrosis progression [100,101,102,103,104]. The reduced release of TGF-β1 was accompanied by the decreased expression of TGF-β1 down-stream proteins, including MMP-9 (matrix metalloproteinase 9), TIMP-1 (tissue inhibitor of metalloproteinase 1), α-SMA (alpha-smooth muscle actin), and finally collagen I and fibronectin (ECM components) [9]. Therefore, juglanin could be used to improve tissue repair by alleviating pulmonary inflammation and fibrosis. In vitro experiments on human lung fibroblasts and mouse epithelial cells indicated that the mechanism behind observed juglanin activity appears to involve the blockage of STING (stimulator of interferon genes) signaling; however, the relationship between STING and TGF-β1 might not be direct, and further studies are needed in this regard. What seems to also be important is that juglanin administration to healthy mice did not affect tested factors and did not interfere with physiological processes (e.g., cellular functions regulated by TGF-β family) but only diminished pathologically enhanced proteins levels. Hence, juglanin might be considered an attractive therapeutic strategy against pulmonary fibrosis and inflammatory lung injury. This conclusion was verified in a mouse model of acute lung injury caused by lipopolysaccharide [80]. Herein, the number of immune cells (neutrophils, lymphocytes, macrophages, eosinophils) and immunoglobulins (IgA, IgE) was reduced, together with the levels of numerous cytokines/chemokines (IL-1β, IL-6, IL-4, IL-17, IL-18, TNF-α, eotaxin) and fibrosis markers (TGF-β1, α-SMA, collagen type I and III). The NF-kB signaling pathway was proved to be involved in the juglanin-ameliorated inflammatory response, and the same was confirmed in vitro in human lung cells. For detailed study parameters and results, see Table 3.

The anti-fibrotic potential of juglanin was also tested in animal models of chronic kidney disease (kidney fibrosis) and hepatitis caused by fructose (liver fibrosis) [10,86]. In the first study [10], the inflammatory response was reduced by suppressing NF-κB/HDAC3 signaling in kidney (also confirmed by in vitro cellular studies, see Table 3), and the expression of fibrosis-associated genes and factors (e.g., TGF-β1; SMA-α; fibronectin 1; collagen type I alpha 1; collagen type I alpha 2) was decreased. That, together with improving lipid and carbohydrate metabolism (see Section 5.2.), resulted in improved glomerular volume and alleviated edema and exfoliation of glomerular mesangial cells, among others. The attenuation of renal injury and dysfunction was confirmed by testing the levels of kidney damage-related molecules (increased nephrin and podocin levels, decreased KIM-1—kidney damage-related molecule 1) and biochemical markers of kidney condition (decreased serum creatinine, creatinine clearance, urinary albumin-to-creatinine ratio, urinary albumin excretion ratio). In the second study (juglanin effect on liver inflammation and fibrosis) [86], the inhibition of NF-kB activation was through the direct or indirect lowering of fructose-induced TLR4 (toll-like receptor 4) activation (considering the study discussed in Section 5.4, probably indirect through Nrf2 (nuclear factor erythroid 2-related factor 2) signaling), and subsequent inactivation of the MAPK pathway. Consequently, the levels of pro-inflammatory cytokines in the serum and liver (e.g., TNF-α, IL-17, IL-10, IL-35, IL-1β, IL-6, IL-18) and the liver fibrotic score were reduced. For detailed study parameters and results, see Table 3.

Considering the promising results of the studies discussed above, juglanin emerges as a potential therapeutic agent for the prevention and treatment of lung, kidney, or liver fibrosis, with the prospect for further exploration in other organs. Its anti-inflammatory and anti-fibrotic activity is mediated through different pathways associated with NF-kB signaling, TGF-β1, and its related proteins.

### 5.2. Juglanin and Metabolic Syndrome Therapy

**Figure 3:**
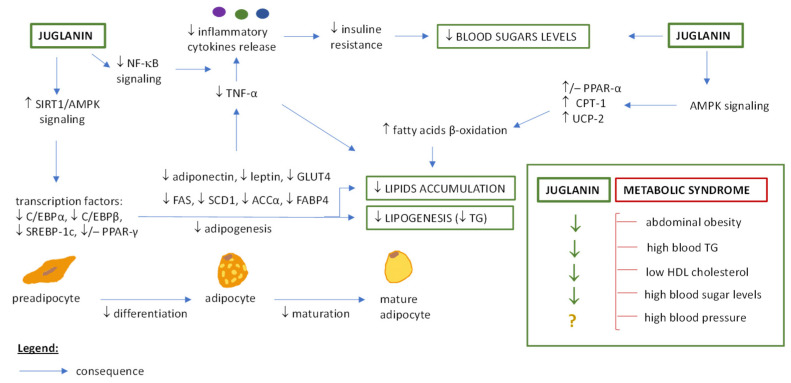
The effects of juglanin on metabolic syndrome. Abbreviations: acetyl-CoA carboxylase α (ACCα); AMP-activated protein kinase (AMPK); carnitine-palmitoyl transferase 1α (CPT-1); CCAAT-enhancer-binding protein α/β (C/EBP α/β); fatty acid synthase (FAS); fatty acid-binding protein 4 (FABP4); glucose transporter 4 (GLUT4); high-density lipoprotein (HDL); nuclear factor-κB (NF-κB); peroxisome proliferator-activated receptor α (PPAR-α); peroxisome proliferator-activated receptor γ (PPAR-γ); sirtuin 1 (SIRT1); stearoyl-CoA desaturase 1 (SCD1); sterol regulatory-element binding proteins 1c (SREBP-1c); triglycerides (TG); tumor necrosis factor-α (TNF-α); uncoupling protein 2 (UCP-2). ↑ increase; ↓ decrease; − not changed.

According to the National Heart, Lung and Blood Institute [105], metabolic syndrome is a cluster of three or more factors, including abdominal obesity, high blood pressure, high blood triglycerides, low HDL (high-density lipoprotein) cholesterol, and high blood sugar levels that together significantly increase the risk of stroke, diabetes, coronary heart disease, and other severe conditions. As the reports discussed below indicate, juglanin has positively affected four of these factors, with one remaining to be tested in the future (Figure 3).

In animal models of kidney and liver diseases induced by a high-fat diet or fructose, juglanin has been revealed to reduce body fat, inhibit lipid accumulation in tested tissues, decrease the levels of serum/blood triglycerides, total cholesterol, LDL (low-density lipoprotein) cholesterol, glucose and insulin, and increase the level of HDL cholesterol [10,80,87]. The improvement of lipid metabolism was through the reduced expression of transcription factors and enzymes responsible for fatty acids synthesis and deposition (i.e., sterol regulatory element-binding factor 1, SREBF1; fatty acid synthase, FAS; stearoyl-CoA desaturase 1, SCD1; peroxisome proliferator-activated receptor γ, PPAR-γ), and enhanced expression of proteins involved in fatty acid β-oxidation, i.e., transforming of fatty acids to energy (peroxisome proliferator-activated receptor α, PPAR-α; carnitine-palmitoyl transferase 1, CPT-1; uncoupling protein 2, UCP-2) [10]. The anti-inflammatory mechanism (regulation of the NF-κB signaling pathway) was also connected with juglanin hypolipidemic activity and improvement of insulin resistance (positive effects on glucose and insulin tolerance tests) [10,86]. In another study [84], the molecular mechanism behind the inhibitory effects of juglanin on adipogenesis was tested in vitro on the 3T3-L1 preadipocytes. Juglanin significantly reduced the lipid accumulation in differentiated adipocytes (day 8 of the study), and the activation of the SIRT1/AMPK (sirtuin 1/ AMP-activated protein kinase) signaling pathway was increased. This effect was reversed in cells treated with AMPK inhibitor; hence, the inhibitory effects of juglanin on adipogenesis were proved to be mediated by AMPK. Juglanin reduced the expression of C/EBPα, C/EBPβ (CCAAT-enhancer-binding protein α/β), and SREBP-1c (sterol regulatory-element binding proteins 1c), key transcriptional factors involved in adipogenesis and lipogenesis, and decreased the levels of adipocyte markers (fatty acid-binding protein 4, FABP4; glucose transporter 4, GLUT4; adiponectin; leptin). In contrast to the earlier-described study [10], here, juglanin did not affect PPAR-α and PPAR-γ [84], which may or may not be due to different experimental conditions (in vivo/in vitro studies, various tissues and doses, etc.; detailed study parameters and results are in Table 3). Finally, juglanin was proposed to suppress appetite by the favorable binding to 5-HT2C receptor (5-hydroxytryptamine receptor 2C) outside of the known agonist binding pocket (molecular docking studies), which may be helpful in therapy for obesity [85] and warrant further studies.

In conclusion, juglanin has been shown to alleviate metabolic syndrome by regulating different aspects of lipid metabolism, and hence lower abdominal obesity and a high blood triglycerides level, and counteract a low HDL level (three out of five metabolic syndrome factors). As for the reduction of high blood glucose (fourth out of five metabolic syndrome factors), while this may involve the suppression of inflammatory response and dyslipidemia, and therefore indirectly reduce insulin resistance and normalize the glucose level [10,84,87], the exact mechanism behind glucose-lowering activity has not been fully explained yet. The previous studies only indicated that juglanin is a weak inhibitor of α-glucosidase (the enzyme responsible for carbohydrate digestion) [32], and thus juglanin activity may or may not involve glucose metabolism rather than glucose absorption. However, no activity grip point can be ruled out or confirmed so far. Additionally, there is no available data regarding the impact of juglanin on blood pressure (the last out of five metabolic syndrome factors).

### 5.3. Hepatoprotective Potential of Juglanin

**Figure 4:**
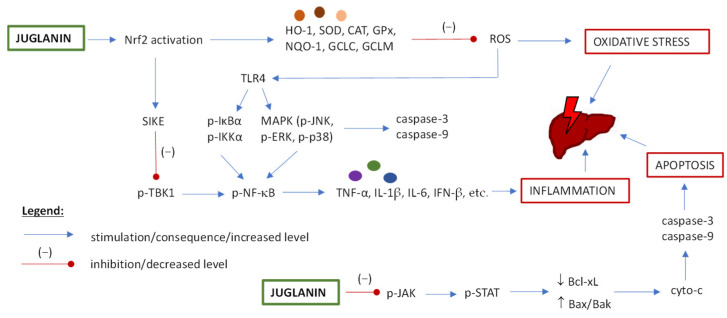
The potential mechanisms of juglanin hepatoprotective activity. Abbreviations: B-cell lymphoma-extra large (Bcl-xL); bcl-2-like protein 4 (Bax); catalase (CAT); glutamate-cysteine ligase subunits (GCLC, GCLM); glutathione peroxides (GPx); interferon β (IFN-β); interleukin 1β (IL-1β); interleukin 6 (IL-6); mitogen-activated protein kinase (MAPK); NAD(P)H quinone dehydrogenase 1 (NQO-1); nuclear factor erythroid 2-related factor 2 (Nrf2); oxygenase 1 (HO-1); phosphorylated signal transducer and activator of transcription (p-STAT); phosphorylated c-Jun N-terminal kinase (p-JNK); phosphorylated extracellular signal-regulated kinase (p-ERK); phosphorylated inhibitor κB-α (p-IκBα); phosphorylated IκB kinase α (p-IKKα); phosphorylated Janus Kinase 2 gene (p-JAK2); phosphorylated nuclear factor-κB (p-NF-κB); phosphorylated TANK-binding kinase 1 (p-TBK1); toll-like receptor 4 (TLR4); reactive oxygen species (ROS); superoxide dismutase (SOD); suppressor of IKKepsilon (SIKE); tumor necrosis factor-α (TNF-α).

The hepatoprotective potential of juglanin is associated with antioxidant, anti-inflammatory and anti-apoptotic activity on hepatic cells (based on in vivo animal studies and in vitro cellular models) [11,86,87]. For a graph of juglanin hepatoprotective mechanisms (discussed below), see Figure 4.

In a mouse model of non-alcoholic steatohepatitis-like phenotype induced by air pollution, juglanin exhibited anti-inflammatory and antioxidant effects via the activation of Nrf2/SIKE (nuclear factor erythroid 2-related factor 2/suppressor of IKKepsilon) signaling pathway [11]. As a consequence, the expression of pro-inflammatory cytokines (IL-1β, IL-6, TNF-α) and IFN-β (interferon β) was reduced, and the expression and/or activity of antioxidant enzymes (e.g., oxygenase 1, HO-1; superoxide dismutase, SOD; NAD(P)H quinone dehydrogenase 1, NQO-1; glutamate-cysteine ligase subunits, GCLC and GCLM) was increased. This mechanism was confirmed in the in vitro cellular study on human liver cells L02 [11]. Moreover, in an animal model of hepatitis caused by fructose, the anti-inflammatory and anti-apoptotic activity of juglanin was through suppression of MAPK and NF-κB signaling, as well as JAK2/STAT3-regulated (Janus kinase gene/signal transducer and activator of transcription) caspase-3 and caspase-9 release [86]. Therefore, juglanin may act as a hepatoprotective agent by affecting different signaling pathways; however, not all possibilities have been verified yet. Nevertheless, the reduction of the serum and liver levels of ALT, AST, ALP (alkaline phosphatase) demonstrated in various studies [10,11,86,87] suggests that juglanin can alleviate hepatic injury and restore liver function. For detailed study parameters and results, see Table 3.

### 5.4. Effects of Juglanin on UVB-Induced Skin Injury, Wound Healing and Cellular Senescence

UVB irradiation produces various harmful responses, including sunburn, edema, hyperplasia, cellular senescence and skin diseases. The simultaneous UVB irradiation and juglanin supplementation of female SKH-1 hairless mice proceeded with decreased skin injury (lower skin thickness and transepidermal water loss), as well as reduced wrinkle formation (lower total groove volume, wrinkle area, wrinkle volume and the number of wrinkles) [12]. For detailed study results, see Table 3.

Among molecular mechanisms associated with UVB-induced skin injury are oxidative stress and inflammation, which were investigated as grip points of juglanin activity. In the course of the study, it was proved that juglanin protects against skin injury by reducing the Nrf2-dependent generation of ROS (reactive oxygen species). Nrf-2 activation is followed by the enhanced activity of antioxidant enzymes (e.g., SOD; CAT, catalase; GPx, glutathione peroxides) and hence the lower level of ROS, which next prevents the MAPKs and NF-κB phosphorylation and the release of pro-inflammatory mediators. Crucially, Nrf2 knockdown (tests on human epidermal cells) did not affect the levels of MAPKs (phosphorylated mitogen-activated protein kinase 38, p-p38; phosphorylated extracellular signal-regulated kinase 1 or 2, p-ERK1/2; phosphorylated c-Jun N-terminal kinase, p-JNK) and p-NF-κB, and consequently COX-2 (cyclooxygenase 2), IL-1β and TNF-α. Therefore, the juglanin effects on MAPKs and NF-κB observed in this and probably other studies seem indirect and Nrf2-dependent [12]. On the other hand, there is still the question of whether the dependence between juglanin and Nrf2 is direct only, or also indirect? The latter possibility is encouraged by the study of Wang et al. [91], in which SIRT1 (an upstream kinase of Nrf2) was the essential factor for juglanin activity. However, at this research stage, the direct effect of juglanin on Nrf2 cannot be completely ruled out.

The ability of juglanin to lower the intracellular level of ROS was also suggested to be responsible for the inhibition of cellular senescence observed in human dermal fibroblasts [78]. In the study, juglanin exerted inhibitory activity against stress-induced (by adriamycin) premature senescence, as well as replicative senescence (when cells reach an irreversible stage of cell cycle arrest following multiple rounds of replication), and the activity of senescence-associated beta-galactosidase (SA-β-gal) was reduced in both cases. On the other hand, no effect on human umbilical vein endothelial cells was observed, suggesting that the impact of juglanin on cellular senescence might be cell-dependent with a specific regulatory target. Still, recognizing an exact mechanism of juglanin activity requires further studies. Nevertheless, juglanin may be a valuable candidate for developing herbal drugs or cosmetics to modulate tissue aging or aging-related diseases.

Finally, juglanin enhanced the percentage of wound closure in the in vitro scratch-wound healing assay on human dermal fibroblasts [15], by promoting the migration of fibroblasts. While the molecular mechanism behind this was not investigated, other studies indicated the engagement of similar pathways to those activated after UVB-induced skin injury discussed above, i.e., through MAPKs inhibition [106].

### 5.5. Juglanin and Cardiovascular Disorders

**Figure 5:**
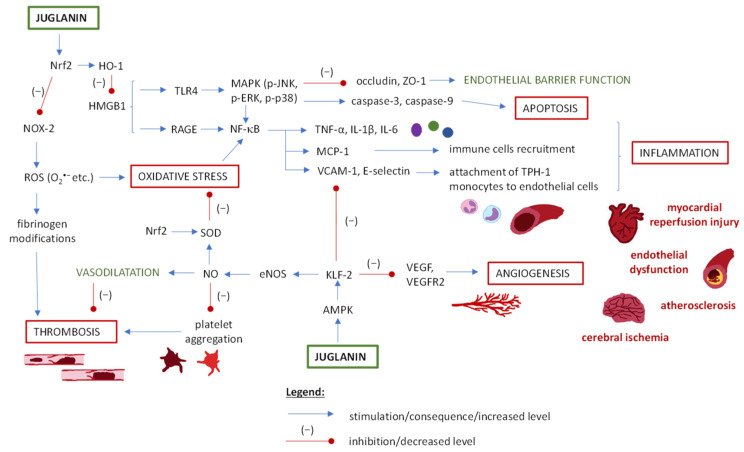
The potential mechanisms of juglanin activity in the cardiovascular system. Abbreviations: AMP-activated protein kinase (AMPK); endothelial nitric oxide synthase (eNOS); high mobility group box 1 (HMGB1); interleukin 1β (IL-1β); interleukin 6 (IL-6); kruppel-like factor 2 (KLF-2); mitogen-activated protein kinase (MAPK); monocyte chemoattractant protein 1 (MCP-1); NADPH oxidase 2 (NOX-2); nitrogen oxide (NO); nuclear factor erythroid 2-related factor 2 (Nrf2); nuclear factor-κB (NF-κB); oxygenase 1 (HO-1); phosphorylated c-Jun N-terminal kinase (p-JNK); phosphorylated extracellular signal-regulated kinase (p-ERK); reactive oxygen species (ROS); receptor for advanced glycation end products (RAGE); superoxide dismutase (SOD); toll-like receptor 4 (TLR4); tryptophan hydroxylase-1 (TPH-1); tumor necrosis factor-α (TNF-α); vascular cellular adhesion molecule-1 (VCAM-1); vascular endothelial growth factor (VEGF); vascular endothelial growth factor receptor 2 (VEGFR2); zonula occludens-1 (ZO-1).

Cardiovascular diseases are a group of disorders of the heart and blood vessels that are the leading cause of death globally [107]. They include diseases of the blood vessels supplying the heart muscle or brain, peripheral arterial disease, rheumatic heart disease, etc. Considering the studies discussed below, juglanin may be helpful in several disorders and conditions within the cardiovascular system, and its effectiveness is based mainly on anti-inflammatory, anti-apoptotic and antioxidant activity, as well as regulation of endothelial permeability and angiogenesis (Table 3, Figure 5) [7,79,88].

In the shear stress-induced endothelial dysfunction (atherosclerosis model) tested on human aortic endothelial cells (HAECs) [79], juglanin treatment resulted in the downregulation of NOX-2 (NADPH oxidase 2) and reduction of intracellular ROS levels. This fact, and the enhancement of the expression and/or activity of antioxidant enzymes (e.g., SOD, CAT, HO-1) observed in another study [11], explains the antioxidant potential of juglanin and its diminishing effects on the harmful consequences resulting from oxidative stress. On the other hand, the multiple in vitro tests on the ROS scavenging potential of juglanin showed a relatively weak anti-radical effect, unlike that observed for, e.g., quercetin, which results from the chemical structure of juglanin (single -OH group in the flavonoid B ring) [90]. Therefore, the mechanism of juglanin activity in oxidative stress is based on antioxidant and pro-oxidant enzyme control rather than direct ROS scavenging.

Oxidative stress is closely linked with inflammation, apoptosis, and thrombosis, leading to endothelial dysfunction, atherosclerosis, myocardial injury, cerebral ischemia, etc. The inhibition of inflammatory response (decreased IL-6, IL-1β, TNF-α, and increased IL-10 levels) and apoptosis (decreased caspase-3 and caspase-9 levels) after juglanin treatment was observed, e.g., in a mouse acute myocardial infarction model, and the regulation of the MAPK signaling pathway was suggested as a responsible molecular mechanism [7]. Based on other studies (see, e.g., Section 5.4), the inhibition of MAPK is probably indirect—through Nrf2 activation and subsequent oxidative stress control [12,86]. All these effects resulted in the improved morphology of myocardial tissue (decreased loose, edematous, and necrotic cardiomyocytes, lower inflammatory cell infiltration) and the decreased CK-MB level (creatine kinase myocardial band, an enzyme released into the blood when heart muscle cells are damaged).

Similar signaling pathways may have been involved in the study of the cellular model of atherosclerosis (HAECs cells), in which the suppressed expression of HMGB1 (high-mobility group box 1) was observed [79]. HMGB1 is a nuclear DNA-binding protein that activates innate immunity, and itself can be inhibited by Nrf2-triggering. Hence, the lowering of HMGB1 secretion may be both indirect and direct (the latter needs to be further studied). Nowadays, inhibiting HMGB1 translocation from the nucleus to the cytoplasm or its direct neutralization is considered a promising approach for cardiovascular treatment. HMGB1 regulates diverse downstream effectors by interacting with its receptors, such as RAGE (receptor for advanced glycation end products) or TLR4. It leads to the release of inflammatory cytokines and chemokines, immune cell recruitment (via, e.g., monocyte chemoattractant protein 1, MCP-1, action) and their attachment to endothelial cells (via, e.g., vascular cellular adhesion molecule-1, VCAM-1, and E-selectin release) [108]. In the study of juglanin, the over-expression of the above-mentioned inflammatory mediators was successfully diminished, making juglanin a promising agent for further studies [79].

Another signaling pathway through which juglanin acts as an anti-inflammatory, antioxidant, and anti-thrombotic agent is with the participation of KLF-2 (Kruppel-like factor 2), a transcription factor engaged in the regulation of vascular endothelial cells metabolism [109]. Juglanin was able to rescue shear stress-induced reduction of KLF-2 expression in aortic endothelial cells, which enhanced eNOS (endothelial nitric oxide synthase) and NO (nitrogen oxide) levels [79]. NO plays a crucial role in regulating vascular tone, affects leukocyte adhesion to the vascular endothelium (via inhibiting NF-κB signaling), is a potent inhibitor of platelet activation, and thus exhibits an anti-thrombotic effect. While the direct test of juglanin activity on human platelets (ex vivo study) showed a weak or no effect on platelet aggregation [4], endothelial NO release seems the preferred mechanism. Moreover, juglanin was proven to prevent oxidative modifications of biomolecules, e.g., fibrinogen (ex vivo studies on human plasma or isolated protein), which also prevents endothelial damage, abnormal fibrin clot and related cardiovascular disorders [89].

Finally, in an animal model of cerebral ischemia, juglanin was found to reduce blood-brain barrier permeability, by enhancing occludin and ZO-1 (zonula occludens-1) protein levels, directly or indirectly (through, e.g., Nrf2/MAPK signaling). The inhibition of VEGF/VEGFR2 (vascular endothelial growth factor/vascular endothelial growth factor receptor 2), and hence reduction of the angiogenesis progression, was also demonstrated, which might be associated with KLF-2 activation or another, still not tested, pathway [88].

Nevertheless, juglanin’s activity in the cardiovascular system is consequential and multi-faceted, but further investigations of molecular mechanisms and pathways are still required. For detailed study parameters and results discussed above, see Table 3, and for a graph of potential molecular targets, see Figure 5.

### 5.6. Juglanin and Central Nervous System Disorders

According to data provided by an integrated online platform for comprehensive predictions of ADMET (adsorption, distribution, metabolism, elimination, and toxicity) properties [66] discussed in detail in Section 3.1, the probability for juglanin to cross the blood-brain barrier (BBB) is low. However, several animal studies indicated that juglanin may protect brain tissue against chemotherapy-induced toxicity and ameliorate depression-like behavior in chronic unpredictable mild stress, as well as attenuate the cognitive dysfunction in Parkinson’s disease [6,76,83]. The detailed methodology and results of these studies (described below) are included in Table 3.

The memory and cognitive function of mice or rats were tested based on different behavior tests (depending on the study), including the Morris water maze test, forced swimming test, space exploration test, Y-maze test, tail suspension test, and open-field test. They indicated an improvement in brain function after juglanin treatment in disorder-induced animals, and on the other hand, no differences were observed when juglanin was given to healthy animals. Therefore, it is apparent that juglanin does not affect the properly functioning nervous system, but only counteracts the malfunctions resulting from diseases, intoxications or other conditions. The normalization of nervous system activity was noticed as, e.g., decreased latency of escapes, immobility time, and the time needed to find a hidden platform, as well as increased time spent in the target quadrant, the frequency of appearance in the target quadrant, higher number of platform crossings, and extended swimming and climbing time [6,76,83].

Histopathological observations of the brain tissue showed that juglanin can alleviate alterations such as pyknosis, degenerated and swollen neurons or congested blood vessels, caused by, e.g., doxorubicin or stress conditions [6,83], and several mechanisms were tested for this effect.

Juglanin was proven to activate the AMPK pathway [6] and inhibit TLR4/MyD88/CD14 signaling [76] in the hippocampus, both of which result in the arresting of NF-κB signaling and enhancement of neuronal survival through the inhibition of neuroinflammation. During inflammation response, the activation of resting microglia and astrocytes occurs (evidenced by, e.g., up-regulated GFAP, glial fibrillary acidic protein, and Iba1, ionized calcium-binding adaptor molecule 1 levels) and pro-inflammatory cytokines and other mediators are released. These effects were reversed by juglanin treatment [6,76,83].

Glial activation also decreased the BDNF level (brain-derived neurotrophic factor), which is responsible for neuronal differentiation and maturation, among others [110]. Juglanin-derived AMPK triggering and the restoration of the proper BDNF level affected the PI3K/AKT (phosphoinositide 3-kinase/protein kinase B) signaling, and via inhibition of caspase and PARP cleavage, it revealed an anti-apoptotic effect [6].

The antioxidant properties of juglanin were also involved in its neuro-protective effects [83]. Oxidative stress in the brain tissue of doxorubicin-treated rats was ameliorated by juglanin, as evidenced by the decreased MDA (malondialdehyde) level. This effect was possibly related to the increased activity of antioxidant enzymes, i.e., SOD and CAT.

In an LPS-stimulated animal model of Parkinson’s disease, it was observed that juglanin treatment increased the Aβ (beta-amyloid), p-Tau (phosphorylated microtubule-associated protein), and α-synuclein expression and protein levels in the hippocampus [76]. The excessive accumulation of these proteins, considered one of the main causative factors of several neurodegenerative disorders (e.g., Alzheimer’s and Parkinson’s diseases), has been associated with toxic effects on neurons, including the induced inflammatory activation of glia, enhanced oxidative stress in neuronal cells, and pro-apoptotic effects [76,111,112]. While the exact mechanisms behind juglanin effects on Aβ, p-Tau, and α-synuclein proteins are still unknown, this type of activity is attention-worthy and a promising direction for further research.

According to the study of Ren et al. [6], juglanin may also affect the serum and hippocampal levels of depression-related neurotransmitters, i.e., increase the levels of 5-HTP (5-HTP5-hydroxytryptophan), 5-HT (5-hydroxytryptamine), DA (dopamine) and GABA (gamma-aminobutyric acid), and decrease the levels of CORT, ACTH, and glutamate. However, whether the normalization of neurotransmitter levels results from the mechanisms described above, or if juglanin may affect them directly (and the involved mechanisms), remains to be tested.

**Figure 6:**
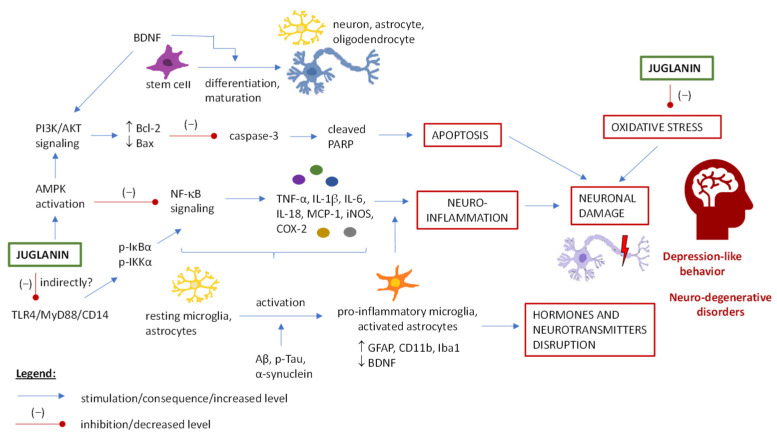
The potential pathways of juglanin activity in the central nervous system. Abbreviations: AMP-activated protein kinase (AMPK); B-cell lymphoma 2 (Bcl-2); Bcl-2-like protein 4 (Bax); beta-amyloid (Aβ); cluster of differentiation 11b (CD11b); cluster of differentiation 14 (CD14); cyclooxygenase 2 (COX-2); enhanced neurotrophic factor (BDNF); glial fibrillary acidic protein (GFAP); inducible nitric oxide synthase (iNOS); interleukin 18 (IL-18); interleukin 1β (IL-1β); interleukin 6 (IL-6); ionized calcium-binding adaptor molecule 1 (Iba1); monocyte chemoattractant protein 1 (MCP-1); myeloid differentiation primary response 88 (MyD88); nuclear factor-κB (NF-κB); phosphoinositide 3-kinase (PI3K); phosphorylated inhibitor κB-α (p-IκBα); phosphorylated IκB kinase α (p-IKKα); phosphorylated microtubule-associated protein (p-Tau); poly-ADP-ribose polymerase (PARP); protein kinase B (AKT); toll-like receptor 4 (TLR4); tumor necrosis factor-α (TNF-α).

Figure 6 summarizes the pathways described above, which were tested as responsible for the action of juglanin in the central nervous system. Activity mechanisms investigated in animal models were also analyzed in vitro in cellular studies on mice primary astrocytes, murine microglial cells, and mouse hippocampal cells (see Table 3). These studies allowed the authors to confirm some of the above-mentioned mechanisms and draw more in-depth conclusions. Eventually, the effects of juglanin on pro-inflammatory mediators release were reversed in the mouse hippocampal cells with silenced AMPK, which suggests that the anti-inflammatory capacity of juglanin is AMPK-dependent [6]. Moreover, the observed inhibition of TLR4 was probably by an indirect route—through Nrf2 signaling [12].

All in all, more attention should be given to investigating the influence of juglanin on the central nervous system. While anti-inflammatory mechanisms seems to be well known for the compound, many other pathways influencing the system balance still need to be explored. For example, is it possible for juglanin to directly influence the activity of neurotransmitters and to what extent, and what is the mechanism of action of juglanin towards proteins associated with neurodegenerative disorders (e.g., amyloid-β)?

### 5.7. Juglanin and Skeletal System Disorders (Arthritis and Osteoporosis)

According to the National Institute of Arthritis and Musculoskeletal and Skin Diseases [113], arthritis literally means the inflammation of a joint, i.e., a place where two bones meet. There are many different types of arthritis differing in cause, location or symptoms. The most common types of arthritis are osteoarthritis (a degenerative joint disease) and rheumatoid arthritis (an autoimmune form).

**Figure 7:**
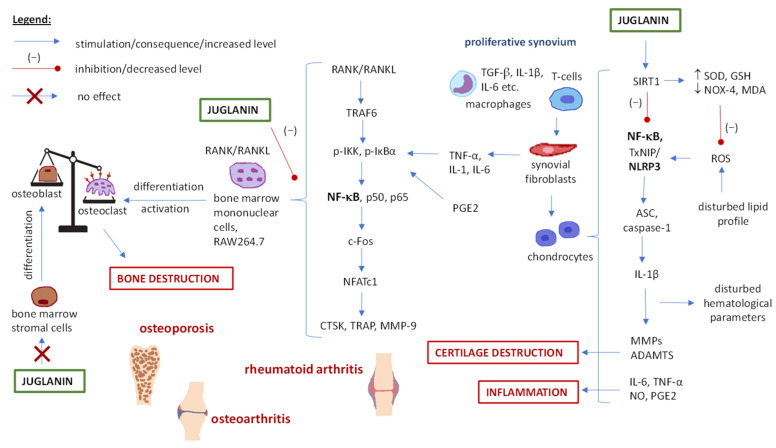
The potential pathways of juglanin activity in the skeletal system. Abbreviations: apoptosis-associated Speck-like protein containing a caspase activation and recruitment domain (ASC); cysteine cathepsin K (CTSK); disintegrin and metalloproteinase with thrombospondin motifs (ADAMTS); factor matrix metalloproteinase (MMP); factor matrix metalloproteinase 9 (MMP-9); glutathione (GSH); interleukin 1 (IL-1); interleukin 1β (IL-1β); interleukin 6 (IL-6); malondialdehyde (MDA); NADPH oxidase 4 (NOX-4); nitrogen oxide (NO); nucleotide-binding domain, leucine-rich–containing family, pyrin domain–containing-3 (NLRP3); nuclear factor-κB (NF-κB); phosphorylated inhibitor κB-α (p-IκBα); phosphorylated IκB kinase (p-IKK); prostaglandin E2 (PGE2); reactive oxygen species (ROS); receptor activator for nuclear factor κ B (RANK); receptor activator for nuclear factor κ B ligand (RANKL); sirtuin 1 (SIRT1); superoxide dismutase (SOD); thioredoxin-interacting protein (TxNIP); transcription factor nuclear factor of activated T cells c1 (NFATc1); Fos proto-oncogene (c-Fos); transforming growth factor β (TGF-β); translating ribosome affinity purification (TRAP); tumor necrosis factor receptor-associated factor 6 (TRAF6); tumor necrosis factor-α (TNF-α).

Studies of adjuvant-induced arthritis (animal model), as well as in vitro cellular tests on mice chondrogenic cells and primary human osteoarthritis chondrocytes, indicated that juglanin may be used as a therapeutic agent for osteoarthritis or rheumatoid arthritis treatment, mainly based on its anti-inflammatory mechanisms [13,91,92] (Table 3, Figure 7). First, it was observed that juglanin suppressed the expression of ADAMTS (disintegrin and metalloproteinase with thrombospondin motifs 4 or 5; ADAMTS-4, ADAMTS-5) and MMPs (factor matrix metalloproteinases 1, 3 or 13; MMP-1, MMP-3, MMP-13) in IL-1β-stimulated chondrocytes [13]. Considering the ADAMTS and MMPs role in the degeneration of cartilage extracellular matrix components (aggrecan and collagen type II), this may protect cartilage against destruction during arthritis. Also, juglanin blocked the production of IL-6, TNF-α, NO, and PGE2 (prostaglandin E2) and downregulated COX-2 and iNOS (inducible nitric oxide synthase) expression, contributing to an overall reduction in inflammation. The proposed mechanism of action was by regulating NF-κB signaling [13]. In another study [91], juglanin was able to mitigate LPS-induced inflammation in chondrocytes by improving the oxidant/antioxidant ratio and inhibiting the activation of the NLRP3 (nucleotide-binding domain, leucine-rich-containing family, pyrin domain-containing-3) inflammasome—a component of the innate immune system responsible for, e.g., IL-1β and IL-18 secretion in response to infection or cellular damage. These effects were achieved through the enhancement of SIRT1 expression, while silencing SIRT1 erased the effects of juglanin on the NLRP3 inflammasome. It is also worth noticing that SIRT1, being the upstream kinase of Nrf2, may be responsible for NF-κB activation [91].

The positive effect of juglanin on oxidant/antioxidant ratio, inflammatory mediators, and ADAMTS was also observed in a rat model of Freund’s complete adjuvant-induced arthritis [92]. Moreover, in this study, juglanin was also able to improve disturbed hematological parameters (increase the levels of red blood cells and hemoglobin, decrease the levels of white blood cells, platelets and erythrocyte sedimentation rate), and lipid profile and hepatic function (lower serum AST, ALT, ALP, and CRP levels), which are common features of rheumatoid arthritis (it may cause medical problems outside of joints). Histopathological analysis of the tibiotarsal joint revealed the attenuation of inflammatory cell infiltration, and cartilage and synovial destruction, and the effect of juglanin at 20–40 mg/kg/day was comparable to that observed after leflunomide treatment (anti-rheumatoid drug) at 10 mg/kg/day (juglanin at 10 mg/kg/day was inactive). Consequently, the symptoms of improvement in pain level and living comfort were observed, i.e., increased paw withdrawal latency and threshold and decreased joint diameter and paw volume.

Another skeletal disorder in which juglanin effectiveness was tested is osteoporosis. This is a bone disease that develops when bone mineral density decreases or when the structure of bone changes, leading to a decrease in bone strength and an increased risk of fractures. Among the causes of osteoporosis are changes to hormones (e.g., low estrogen levels in postmenopausal women), medications, and other medical conditions, such as the development of the autoimmune process and inflammation in the course of rheumatoid arthritis [114].

In a mouse model of bone loss induced by ovariectomy, juglanin reduced the number of osteoclasts (a type of bone cell which breaks down bone tissue), decreased trabecular separation, and increased bone volume and trabecular number [82]. Further in vitro studies on bone marrow mononuclear cells and RAW264.7 macrophages showed that juglanin inhibited RANKL-induced (receptor activator for nuclear factor κ B ligand) osteoclasts differentiation and formation (osteoclastogenesis). The expression of osteoclastogenic genes (of Fos proto-oncogene, c-Fos; tartrate-resistant acid phosphatase, TRAcP; cysteine cathepsin K, CTSK; and MMP-9) and transcription factor NFATc1 (transcription factor nuclear factor of activated T cells c1) was reduced, which was suggested to be through inhibition of the NF-κB signaling pathway. On the other hand, juglanin did not affect bone marrow stromal cell differentiation (i.e., osteoblast formation); hence, it had no effect on the formation of new bone tissue. Therefore, the accumulated research suggests that juglanin treatment may stop the development and progression of osteoporosis rather than reverse the damage already done to the bones.

For a comprehensive overview of the parameters and results discussed above, refer to Table 3, and for a graphical representation of the pathways targeted, see Figure 7.

### 5.8. Juglanin and Anti-Cancer Potential

**Figure 8:**
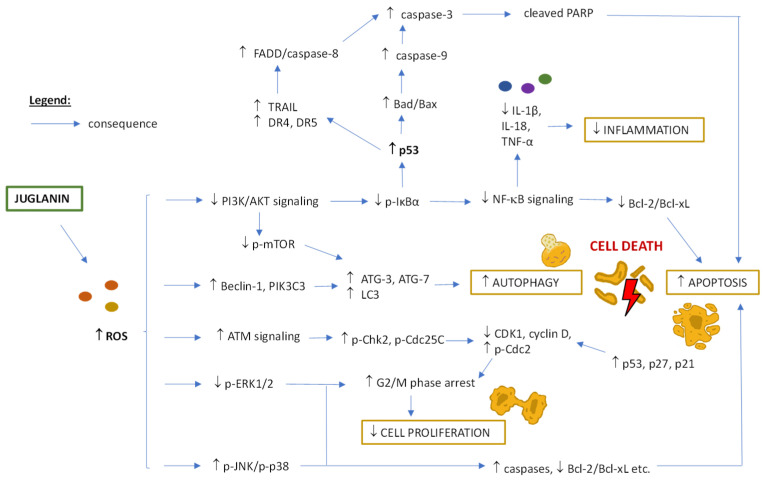
The potential metabolic pathways of juglanin anti-cancer activity. Abbreviations: ataxia telangiectasia mutated (ATM); autophagy protein 7, 3 (ATG-7, ATG-3); B-cell lymphoma 2 (Bcl-2); B-cell lymphoma-extra large (Bcl-xL); bcl-2-like protein 4 (Bax); BH3 domain-containing protein (Bad); cyclin-dependent kinase 1 (CDK1); death receptor 4 (DR4); death receptor 5 (DR5); FAS-associated death domain protein (FADD); gap 2 phase/mitosis phase in the cell cycle (G2/M); interleukin 18 (IL-18); interleukin 1β (IL-1β); microtubule-associated protein light chain 3 (LC3); nuclear factor-κB (NF-κB); phosphatidylinositol 3-kinase catalytic subunit type 3 (PIK3C3); phosphoinositide 3-kinase (PI3K); phosphorylated cell division cycle 2 (p-Cdc2); phosphorylated cell division cycle 25C (p-Cdc25C); phosphorylated checkpoint kinase 2 (p-Chk2); phosphorylated c-Jun N-terminal kinase (p-JNK); phosphorylated extracellular signal-regulated kinase 1/2 (p-ERK 1/2); phosphorylated inhibitor κB-α (p-IκBα); phosphorylated mammalian target of rapamycin (p-mTOR); poly-ADP-ribose polymerase (PARP); protein kinase B (AKT); reactive oxygen species (ROS); TNF-related apoptosis-inducing ligand (TRAIL); tumor necrosis factor-α (TNF-α).

The anti-cancer potential of juglanin and the related mechanisms were tested in animal models and cellular studies on different cell lines of skin cancer (B16F10 murine melanoma cells), breast cancer (human breast cancer cell lines MCF-7, SKBR3), and lung cancer (cell lines A549, H1975) [8,77,81]. They evidenced that juglanin may trigger cell apoptosis and autophagy and reduce cell proliferation via multiple targeting genes. For a graph of potential metabolic pathways of juglanin anti-cancer activity (discussed below), see Figure 8, and for detailed study parameters and results, see Table 3.

First, juglanin promoted apoptosis in lung cancer cells through increasing p53-dependent activation of TRAIL/DRs (TNF-related apoptosis-inducing ligand/ death receptors) signaling and, consequently, the levels of cleaved PARP and caspase-3 [8]. This effect was reversed with a p53 inhibitor, which reduced the number of apoptotic cells. Among other signaling pathways involved in juglanin pro-apoptotic effects were PI3K/AKT and MAPKs (ERK1/2, JNK, p38), also associated with anti-inflammatory potential (via the inhibition of NF-κB). Interestingly, juglanin-mediated apoptosis was enhanced using PI3K/AKT or ERK1/2 inhibitors and p38 or JNK activators, which suggested the synergistic potential of juglanin with other drugs. Both of these paths may be engaged in the reduction of anti-apoptotic members Bcl-2 (B-cell lymphoma 2) and Bcl-xL (B-cell lymphoma-extra-large), and an increase in the pro-apoptotic members Bax (bcl-2-like protein 4), Bad (BH3 domain-containing protein), and PARP observed in juglanin-treated cells and animals. Also, the expression of autophagy marker LC3 (microtubule-associated protein light chain 3) was enhanced after juglanin administration as a result of the Beclin-1 and PIK3C3 (phosphatidylinositol 3-kinase catalytic subunit type 3) over-expression. Therefore, in this study, juglanin induced both apoptosis (i.e., the process of programmed cell death) and autophagy (i.e., a controlled breakdown of intracellular organelles and chemical molecules), resulting in tumor volume and weight reduction in the mouse xenograft model. However, all possible pathways for juglanin activity still need to be discovered due to the complexity of networks between apoptosis and autophagy. What might be essential is that juglanin’s effects on cell viability and apoptosis were reversed in vitro with ROS scavengers, suggesting the importance of ROS production for juglanin effectiveness. Moreover, the toxic effects of juglanin were highly cancer-targeted, i.e., no impact on normal cells and tissues was observed in neither cellular nor animal models (for details, see Section 4.2 and Section 4.3).

Similar pathways of juglanin anti-cancer impact were observed in other studies, i.e., via JNK, p38, PI3K and AKT signaling in a mouse model of UVB-induced skin cancer [81] and JNK signaling in the breast tumor-transplanted mouse model [77]. This resulted in less intense skin vascularization, reduced epidermal hyperplasia and inflammatory cell infiltration, and enhanced apoptotic cell death in the first study, as well as lower tumor volume and a higher level of apoptotic cells in the second study. In both cases, no harmful effects of juglanin activity were observed on normal cells, while in the in vitro studies on cancerous cells, the pro-apoptotic, pro-autophagy and anti-inflammatory activities were confirmed.

Eventually, juglanin was demonstrated to inhibit the proliferation of breast cancer cells by leading to G2/M (gap 2 phase/mitosis) phase arrest, which was represented by a higher number of cells in the G0/G1 and S phases and a lower number of cells in the G2/M phase [77]. This effect came from the regulation of cell cycle-related proteins, i.e., increased phosphorylation of Chk2 (checkpoint kinase 2), Cdc25C (cell division cycle 25C), and Cdc2 (cell division cycle 2), a higher level of p27, and a lower level of cyclin D observed in cancer cells treated with juglanin [77]. The ability of juglanin to modulate the cell cycle was also confirmed in a mouse model of skin cancer induced by UVB [81].

Therefore, given the anti-cancer effects of juglanin without toxicity to normal cells and tissues, this compound may be considered a promising agent for further investigation in anti-tumor therapy, including finding optimal doses, identifying other toxicity mechanisms, verifying potential synergy with known anti-cancer drugs, and testing juglanin’s effectiveness on a wider panel of cancer cells. As for the second, the screening tests on ovarian cancer cells (SK-OV-3), skin melanoma cells (SK-MEL-2), and colon cancer cells (HCT-15) showed no toxicity after juglanin treatment (IC_50_ > 100 µM based on sulforhodamine B assay) [25]. However, since the same was observed for lung carcinoma epithelial cells (A549), which means a contradictory result to the above-mentioned study of apoptotic and autophagy mechanisms (at 20–40 µM) [8], the possibility of juglanin’s impact on any of the tested cells cannot be excluded. The discrepancy in the cited research may result from differences in some experimental conditions, especially parameters tested, e.g., mitochondrial activity versus protein content, or other unknown reasons.

### 5.9. Juglanin and Antifungal, Antiviral and Antiparasitic Potential

Compared to the diseases discussed above, the antimicrobial potential of juglanin seems to be little known. The antifungal activity of juglanin was tested on nine *Candida albicans* and *Candida parapsilosis* strains, but only for two of them was a moderate effect observed [33]. The MIC (minimal inhibitory concentration) for *Candida albicans* 138 was 16 μg/mL (in contrast to 8 μg/mL of fluconazole), and for *Candida parapsilosis* 11 it was 64 μg/mL (compared to 1 μg/mL of fluconazole). As for the antiparasitic activity, juglanin demonstrated a very weak effectiveness against *Plasmodium falciparum* strain 3D7 compared to an antimalarial drug, i.e., IC_50_ for plasmodium viability was between 24 and 120 μM for juglanin and 0.014 μM for chloroquine [35]. Finally, in the case of antiviral activity (against SARS-CoV-2, influenza virus, and dengue virus 2), most of the results, while promising, are based on computational studies (QSAR analysis and molecular docking studies) [93,94,95,96,97] and require confirmation in vitro and in vivo (for details, see Table 3).

### 5.10. Other Effects

Among other possible effects of juglanin on human health is its ability to inhibit rat lens aldose reductase (in vitro test); however, the lack of positive control in this study makes it impossible to comment on whether this activity is significant. Aldose reductase is an oxidoreductase enzyme that catalyzes the reduction of glucose to sorbitol as the first step in the polyol pathway of glucose metabolism. Hence, its inhibition, besides other effects of juglanin discussed in Section 5.2, like lowering blood glucose level, may be important for treating or preventing diabetes complications. The confirmation of this activity is crucial for verifying the antidiabetic potential of juglanin.

Finally, there is one study of juglanin’s protective activity against hearing loss induced by neomycin, i.e., a highly ototoxic antibiotic [36]. In a test on the wild-type larvae zebrafish, juglanin had a moderate but significant effect on the number of otic hair cells, and in the in vitro cellular study on mouse auditory cells an enhancement of cell viability was observed. While the mechanism behind this property was not tested, it may involve the lowering of ROS production and apoptotic cells death [115].

## 6. Materials and Methods

The literature search was performed based on the Scopus, Web of Science, and PubMed databases, searching for original articles written mainly in English. The search was conducted using the keywords “juglanin” or “kaempferol 3-*O*-α-L-arabinofuranoside” or “kaempferol arabinoside” or “kaempferol 3-*O*-arabinofuranoside” or “kaempferol 3-*O*-arabinoside”. The inclusion or exclusion of the articles was validated manually by reading the entire item. Papers about other compounds, e.g., juglanin C which is a diarylheptanoid and not flavonoid, were excluded from the analysis. The review includes literature dating from 1978 to May 2024. The binomial names of the species mentioned in the review were checked and revised according to World Flora Online [116], and hence they may differ from the names used in the corresponding papers.

## 7. Conclusions

**Figure 9:**
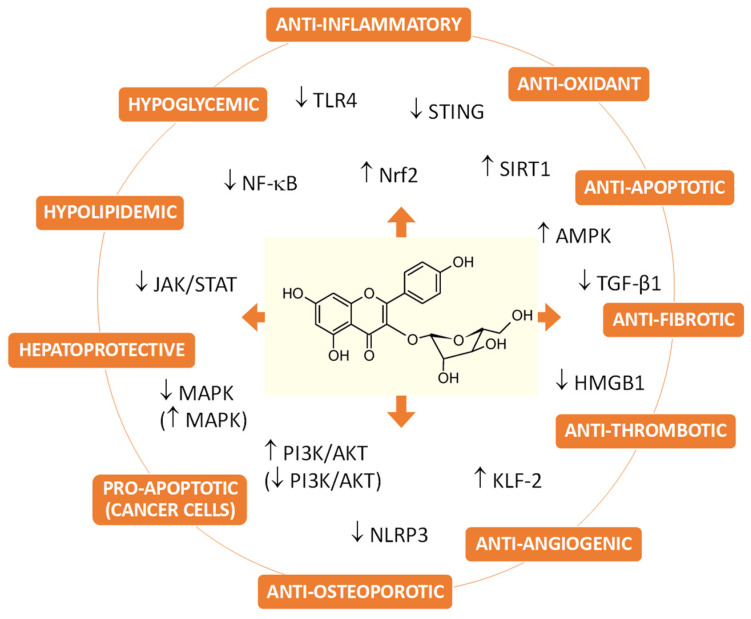
Summary of the potential pathways of juglanin’s biological activity. Abbreviations: AMP-activated protein kinase (AMPK); high mobility group box 1 (HMGB1); Janus kinase gene/signal transducer and activator of transcription (JAK/STAT); Kruppel-like factor 2 (KLF-2); mitogen-activated protein kinase (MAPK); nuclear factor erythroid 2-related factor 2 (Nrf2); nuclear factor-κB (NF-κB); nucleotide-binding domain, leucine-rich-containing family, pyrin domain-containing-3 (NLRP3); phosphoinositide 3-kinase/protein kinase B (PI3K/AKT); sirtuin 1 (SIRT1); stimulator of interferon genes (STING); toll-like receptor 4 (TLR4); transforming growth factor β1 (TGF- β1).

In recent years, different aspects of juglanin’s biological properties have been evaluated, and various signaling pathways, which can influence each other in more or less cross-linked ways, have been indicated as responsible for juglanin’s activity. Juglanin has been proved to affect directly or indirectly a number of functional proteins like Nrf2, STING, NF-κB, SIRT1, AMPK, AKT, MAPK, JAK, NLRP3, TGF- β1, or KLF-2, among others, and thus have anti-inflammatory, antioxidant, anti-fibrotic, anti-thrombotic, anti-angiogenic, anti-osteoporotic, hepatoprotective, hypolipidemic, hypoglycemic, and anti-apoptotic (or in case of cancer cells, pro-apoptotic) effects (Figure 9). Juglanin was suggested to be a promising agent for the prevention or treatment of various diseases or conditions, including: fibrosis, metabolic syndrome, atherosclerosis, cerebral ischemia, myocardial reperfusion injury, skin injury, neurodegenerative disorders, osteoporosis, arthritis, and different types of cancers. Regardless of how much is known, there are still many questions about the exact mechanisms of action and molecular targets for the compound, especially within the central nervous or cardiovascular systems, in what way juglanin can lower blood glucose, and what is its real anti-cancer or antimicrobial potential. Moreover, although the available animal and cellular studies suggest no toxicity of juglanin to normal cells and tissues, this topic should also be investigated in more detail. Last but not least, in the face of promising biological properties and limited plant sources, new origins of the compound and isolation methods should be explored. The high price of juglanin available on the market is a major restriction in broader studies of juglanin and the reason for the lack of clinical trials. Currently, *Prunus serotina* leaves, *Prunus spinosa* flowers, and *Rubus crataegifolius* fruits seem to be the most promising candidates for large-scale and cost-effective isolation of juglanin. Hence, by making juglanin more available, further in vivo research and application studies will become more practical.

## Figures and Tables

**Table 1:** The occurrence of juglanin in the plant kingdom.

Species	Family	Plant Part (Juglanin Content if Determined)	Extract (Juglanin Content if Determined)	Methods	References
*Aesculus hippocastanum* L.	Sapindaceae Juss.	flowers	MeOH (2.96 mg/g dw)	UHPLC–PDA–ESI–TQ–MS/MS, HPLC	[14]
*Afgekia mahidoliae* B. L. Burtt and Chermsir.		leaves	MeOH	isolation, LC-MS, 1D NMR	[15]
*Arbutus unedo* L.		leaves (1.0–1.4 mg/g depending on season, light and precipitation treatment)	50% MeOH	HPLC and LC-MS analysis	[16]
*Bauhinia longifolia* (Bong.) Steud.	Fabaceae Juss.	leaves	EtOH	UHPLC-HR-QTOF-MS	[17]
*Cornus canadensis* L.	Cornaceae Bercht. ex J. Presl	leaves	50% EtOH	isolation, 1D NMR	[18]
*Cupressus duclouxiana* Hickel	*Cupressaceae* Gray	leaves and branches		isolation, NMR	[19]
*Dryopteris lacera* (Thunb.) Kuntze	Dryopteridaceae Herter	leaves	70% EtOH	UPLC-QTOF-MS	[20]
*Euphorbia maculata* L.	Euphorbiaceae Juss.	all plant	MeOH	isolation, LC-MS, 1D NMR	[21,22,23]
		herb (0.24 mg/g dw)	MeOH (0.99 mg/g dw)	HPLC	[24]
*Geranium platyanthum* Duthie	Geraniaceae Juss.	aerial parts	80% MeOH	isolation, FAB-MS, 1D NMR	[25]
*Juglans cinerea* L.	Juglandaceae DC. ex Perleb	leaves	70% EtOH	HPLC, 1D NMR	[2]
*Juglans regia* L.		leaves	50% MeOH (3.23 mg/g dw)	UPLC-PDA-MS/MS	[26]
			70% EtOH	HPLC, 1D NMR	[2]
		flowers	50% EtOH	UHPLC-QTOF-MS	[27]
*Kalanchoe pinnata* (Lam.) Pers.	Crassulaceae J. St.-Hil.	leaves	water	UPLC-OT-FTMS	[28]
*Lespedeza cuneata* (Dum.Cours.) G. Don.	Fabaceae Juss.	leaves	MeOH	isolation, IR, UV, MS, NMR	[29]
*Ludwigia adscendens* (L.) H. Hara	Onagraceae Juss.	aerial parts	CHCl_3_, EtOAc and then MeOH	isolation, UV, LC-MS, 1D and 2D NMR	[30]
*Mallotus nanus* Airy Shaw	Euphorbiaceae Juss.	leaves	MeOH	isolation, LC-MS, 1D and 2D NMR	[31]
*Malus halliana Koehne*	Rosaceae Juss.	leaves	70%EtOH	isolation, LC-MS, 1D NMR	[32]
*Myrcia tomentosa* (Aubl.) DC.	Myrtaceae Juss.	leaves	EtOH	isolation, 1D and 2D NMR, GC/MS	[33]
			EtOAc	isolation, 1D NMR	[34]
*Ozoroa obovata* (Oliv.) R. Fern. and A. Fern.	Anacardiaceae R. Br.	leaves	dichloromethane-methanol (1:1, *v*/*v*)	isolation, LC-MS, 1D NMR	[35]
*Persea americana* Mill.	Lauraceae Juss.	leaves	MeOH	isolation, LC-MS, 1D and 2D NMR	[36]
*Polygonum aviculare* L.	Polygonaceae Juss.	whole plant	70% MeOH	isolation, LC-MS, 1D and 2D NMR	[37]
		herb	50% MeOH	UHPLC–DAD-MS	[38]
			80% MeOH	isolation, LC-MS, 1D NMR	[3]
			EtOH (3.21 mg/g dw), MeOH (3.05 mg/g dw), 70% MeOH (2.42 mg/g dw), 30% MeOH (2.02 mg/g dw)	HPLC	[39]
		leaves	70% EtOH (3.6 mg/g dw)	HPLC	[40]
*Prunus serotina* Ehrh.	Rosaceae Juss.	leaves (0.4–2.2 mg/g dw), inflorescences (0.8–1.6 mg/g dw), depending on the collection time	MeOH	HPLC	[41]
		leaves	70% MeOH	isolation, TLC, UV, 1D NMR	[42]
*Prunus serrulata* Lindl.		leaves	MeOH	isolation, 1D NMR	[43]
*Prunus spinosa* L.		leaves	70% MeOH	isolation, TLC, UV, 1D NMR	[44]
		flowers		isolation, UV, 1D NMR	[45]
			water (8.61 mg/g dw), 70% MeOH (13.73 mg/g dw)	UHPLC-ESI-MS, HPLC	[4]
		flowers (2.60–5.72 mg/g dw depending on the season and source)	70% MeOH	HPLC	[46]
*Rhododendron schlippenbachii* Maxim.	Ericaceae Juss.	leaves	70% Acetone	isolation, NMR	[47]
*Rosa chinensis* Jacq.	Rosaceae Juss.	flowers	70% EtOH	isolation, LC-MS, 1D NMR	[48]
*Rosa damascena* Herrm.		flowers (marc after essential oil distillation)	80% MeOH	isolation, UV, IR, 1D and 2D NMR, ESI-QTOF-MS	[49]
*Rosa rugosa* Thunb.		petals	70% EtOH	UHPLC-Q-orbitrap-HRMS/MS	[50]
		flowers	commercial oral solution (Meiguihua, 23–30 µg/mL)	UHPLC-Q-orbitrap-HRMS/MS, HPLC	[51]
*Rubus cuneifolius* Pursh		ripe fruit (0.05 mg/g dw), unripe fruit (0.39 mg/g dw), leaves (0.68 mg/g dw)	MeOH	HPLC	[5]
*Rubus coreanus* Miq.		ripe fruit (0.11 mg/g dw), unripe fruit (0.40 mg/g dw), leaves (0.54 mg/g dw)			
*Rubus crataegifolius* Bunge		ripe fruit (5 mg/g dw), unripe fruit (1.62 mg/g dw), leaves (0.83 mg/g dw)			
*Rubus pungens var. oldhamii* (Miq.) Maxim.		ripe fruit (0.10 mg/g dw), unripe fruit (n.d.), leaves (0.62 mg/g dw)			
*Rubus rigidus* Sm.		aerial part	EtOH	isolation, 1D and 2D NMR	[52]
*Rumex vesicarius* L.	Polygonaceae Juss.	seeds	water extract	UPLC-QTOF-MS	[53]
*Salvia sclarea* L.	Lamiaceae Martinov	herb	salmus (a side product of the essential oil distillation)	TLC	[54]
*Selliguea hastata* (Thunb.) H. Ohashi and K. Ohashi	Polypodiaceae J.Presl & C.Presl	herb		isolation, NMR	[55]
		whole plant	EtOH	isolation, UV, MS, 1D and 2D NMR	[56]
*Senna obtusifolia* (L.) H. S. Irwin and Barneby	Fabaceae Juss.	leaves	MeOH	isolation, IR, MS, NMR	[57]
*Xylopia parviflora* Spruce	Annonaceae Juss.	fruits	MeOH	isolation, HPLC, LC-MS, NMR	[58]

Abbreviations: dw, dry weight; electrospray ionization quadrupole time-of-flight mass spectrometry (ESI-QTOF-MS); ethanol extract (EtOH); ethyl acetate extract (EtOAc); fast atom bombardment mass spectrometry (FAB-MS); gas chromatography–mass spectrometry (GC/MS); high-performance liquid chromatography (HPLC); infrared spectroscopy (IR); liquid chromatography-high resolution quadrupole-time of flight mass spectrometer method (UHPLC-HR-QTOF-MS); liquid chromatography–mass spectrometry (LC-MS); mass spectrometry (MS); methanol extract (MeOH); one-dimensional nuclear magnetic resonance (1D NMR); rapid ultrahigh performance liquid chromatography–electrospray ionization mass spectrometry (UHPLC-ESI-MS); thin layer chromatography (TLC); two-dimensional nuclear magnetic resonance (2D NMR); ultra-high performance liquid chromatography coupled to photo diode array and electrospray ionization mass and triple quadrupole tandem mass spectrometry (UHPLC–PDA–ESI–TQ–MS/MS); ultra-high performance liquid chromatography coupled to diode array detection and mass spectrometry (UHPLC–DAD-MS); ultra-high performance liquid chromatography–quadrupole time-of-flight–mass spectrometry analysis (UPLC-QTOF-MS); ultra-high-performance liquid chromatography coupled to quadrupole-orbitrap high resolution mass spectrometry (UHPLC-Q-orbitrap-HRMS/MS); ultra-high-performance liquid chromatography-hybrid quadrupole time-of-flight mass spectrometry (UHPLC-QTOF-MS); ultraperformance liquid chromatography fusion orbitrap mass spectrometry (UPLC-OT-FTMS); ultraviolet (UV).

**Table 2:** Cellular safety of juglanin measured on human or mice cells (normal or immortalized, but non-tumorigenic).

Cells	Concentration Tested	Safe Concentration	Method	References
mice lung epithelial cells MLE 12	2.5, 5, 10, 20, 40, 80, 160 μM	2.5–160 μM	MTT, 48h	[9]
mice primary astrocytes from 1- to 3-day-old neonatal SD mice	10, 20, 40, 80, 160 μM	10–160 μM	MTT, 72h	[76]
mice hippocampal cells line HT-22	2.5–100 μM	2.5–100 μM	CCK-8, 24h	[6]
	40 μM	40 μM	CCK-8, 72h	
human breast cells HCC1937	2.5, 5, 10, 20, 30, 40 μM	5–20 μM (at 40 μM viability about 70%)	MTS, 24 h, 48 h	[77]
human breast cells HGC-27		5–30 μM (at 40 μM viability about 80%)		
human umbilical vein endothelial cells	24 μM	24 μM	MTT, 24 h	[78]
human aortic endothelial cells	0.25–5 and 25–50 μM	0.25–5 μM (at 50 μM viability about 75%, at 25 μM about 85%)		[79]
human liver cell line L02	5, 10, 20, 40, 80 μM	5–80 μM	MTT, CCK8, LDH release, 24 h	[11]
	40 μM	40 μM	MTT, CCK8 and LDH, 72 h	
	1.25, 2.5, 5, 10, 20, 40, 80 μM	1.25–80 μM	CCK-8, 24 h	[80]
			MTT, 24 h	[8]
human lung cells line BEAS-2B			CCK-8, 24 h	[80]
human lung cells line MRC-5	2.5, 5, 10, 20, 40, 80, 160 μM	2.5–80 μM (at 160 μM viability about 80%)	MTT, 24 h	[9]
	1.25, 2.5, 5, 10, 20, 40, 80 μM	1.25–80 μM		[8]
human kidney cells line HK2				
	1.25, 2.5, 5, 10, 20, 40, 80, 100 μM	1.25–100 μM		[10]
	40 μM	40 μM (ranging from 0 to 72 h, after 96h about 80% viability)	MTT, 96 h	
human dermal fibroblasts CCD-1064sk	1, 5, 10, 20, 40 and 80 μM	1–20 μM	MTT, 24 h	[15]
human dermal fibroblasts	24 μM	24 μM		[78]
human foreskin fibroblasts Hs68	2.5, 5, 10, 20, 30 μM	2.5–30 μM	MTS, 24 h	[81]
human bone marrow stromal cells (BMSCs)	10, 20, 40, 80, 160, 320, 640 μM	10–80 μM (at 160 μM viability about 80%)	MTT, 24 h	[82]
primary human chondrocytes culture	10, 20, 40, 80 μM	10–40 μM (at 80 μM viability about 90%)		[13]
human microglial cells line BV2	2.5–250 μM	2.5–250 μM	CCK-8, 24 h	[6]
	40 μM	40 μM	CCK-8, 84 h	
human keratocytes HaCaT	5, 10, 20, 40, 80, 160 μM	5–160 μM	MTT, 24 h	[12]

Abbreviations: cell counting Kit-8 (CCK-8) based on water-soluble tetrazolium salts; lactate dehydrogenase (LDH); (3-(4,5-dimethylthiazol-2-yl)-5-(3-carboxymethoxyphenyl)-2-(4-sulfophenyl)-2H-tetrazolium) assay (MTS assay); 3-(4,5-dimethylthiazol-2-yl)-2,5-diphenyltetrazolium bromide assay (MTT assay).

**Table 3:** Biological activity of juglanin.

Disease/Pathology Model	Activity Tested, Type of Study	Type of Groups, Treatment	Effects of Juglanin Treatment	Reference
**Discussed mainly in** Section 5.1**: Juglanin and fibrosis treatment**				
idiopathic pulmonary fibrosis (IPF) induced by bleomycin	anti-inflammatory, anti-fibrotic; in vivo animal model; male C57BL/6J mice; n = 20/group	1. normal control 2. juglanin control 3. bleomycin control 4. bleomycin and juglanin (80 mg/kg/day p.o.), duration 21 days	↑ survival rate; ↓ lung injury (↓ neutrophil alveolar infiltration and lung injury score; ↓ protein levels in BALF; ↓ neutrophils and macrophages in BALF; ↓ MPO activity; ↓ expression level of cytokines (IL-6), chemokines (CXCL1) and TNF-α in BALF and lung); ↓ pulmonary fibrosis (↓ fibrotic area in lungs; ↓ collagen I, fibronectin, TIMP-1, MMP-9, α-SMA and TGF-β1 levels in lung); ↓ expression of STING group 4 compared to group 3 for all results; no effect of juglanin on healthy mice	[9]
	anti-inflammatory, anti-fibrotic (late stage of fibrosis); in vivo animal model; male C57BL/6J mice; n = 20/group	1. normal control 2. bleomycin control 3. bleomycin with juglanin (80mg/kg p.o.) from 10th day of bleomycin administration, for 12 days	↑ survival rate; ↓ histological changes in pulmonary tissues and lung injury score; ↓ neutrophils in BALF; ↓ level of IL-6 and TNF-α; ↓ collagen accumulation; ↓ expression levels of TGF-β1, fibronectin, α-SMA, collagen I, and STING group 3 compared to group 2 for all results	
pulmonary fibrosis	anti-fibrotic; in vitro cellular study; the human lung fibroblast cell line MRC-5	1. normal control 2. cells with TGF-β1 induced pathology 3. cells with juglanin (40 μM) and TGF-β1	↓ expression of STING, fibronectin, MMP-9, α-SMA and collagen I group 3 compared to group 2 for all results	
	anti-inflammatory, anti-fibrotic; in vitro cellular study; mouse lung epithelial cells MLE 12	1. normal control 2. cells with bleomycin induced pathology 3. cells with juglanin (40 μM) and bleomycin	↓ mRNA expression levels of TNF-α and IL-6; ↓ expression of STING, TGF-β1 and α-SMA group 3 compared to group 2 for all results	
acute lung injury caused by lipopolysaccharide	anti-inflammatory, anti-fibrotic; in vivo animal model; male C57BL6 mice; n = 10/group;	1. normal control 2. LPS-treated group 3–4. LPS-treated group + juglanin (10 or 20 mg/kg p.o.), duration 3 weeks	↓ inflammation scores, PAS positive cell levels, and fibrosis level (Sirius red positive cells); ↓ total number of neutrophils, lymphocytes, macrophages, eosinophils, and the total number of immune cells in BAL; ↓ eotaxin (chemokine) levels in BAL; ↓lgE and IgA levels in serum and BAL; ↓ fibrosis (↓ TGF-β1, α-SMA, collagen type I and III mRNA levels); ↓levels of IL-1β, IL-6, IL-4, IL-17, IL-18, TNF-α in the serum and lung tissue; effect on IKKα/ NF-κB signaling pathway (↓ p-IKKα, p-NF-κB, IκBα, mature IL-1 β levels) groups 3 and 4 compared to group 2 for all results	[80]
lung injury	anti-inflammatory, anti-fibrotic; in vitro cellular study; the human lung cell line BEAS-2B	1. normal control 2. cells with LPS induced pathology 3. cells treated with LPS and juglanin (40 or 80 μM)	↓ α-SMA *, Collagen I *, Collagen III *, TGF-α * levels (suppression of fibrosis markers); effect on IKKα/ NF-κB signaling pathway (↓ p-IKKα, p-NF-κB *, IκBα *, mature IL-1 β * levels) group 3–5 compared to group 2 for all tests; * juglanin at 40–80 μM; juglanin at 80 μM for other results	
**Discussed mainly in** Section 5.1**: juglanin and fibrosis treatment**; **and** Section 5.2**: juglanin and metabolic syndrome therapy**				
metabolic syndrome and chronic kidney disease (CKD) induced by high fat diet	anti-inflammatory, anti-fibrotic, hypolipidemic, anti-diabetic; in vivo animal model; male C57BL/6 mice; n = 4–12/group	1. normal chow diet group 2. high fat diet (HFD) group 3–5. HFD and juglanin (7.5, 15 or 30 mg/kg/day p.o.), duration 16 weeks	↓ body weight, weight gain in inguinal fat pad, serum TG, TC, LDLC (juglanin 15–30 mg/kg vs. HFD group); ↓ serum ALT *, AST *, and uric acid *; ↓ fasting blood glucose, fasting insulin, HOMA-IR, improved GTT and ITT parameters (↓ AUC); ↓ kidney weight; ↓ BUN, serum creatinine, creatinine clearance, UACR, UAER, collagen accumulation; improved glomerular volume, proliferated mesangial matrix, alleviated the edema and exfoliation of glomerular mesangial cells; ↓ expression of TGF-β1, SMA-α, CTGF, FN1, COL1A1 and COL1A2 (fibrosis associated genes); ↑ nephrin and podocin levels, ↓ KIM-1 (kidney damage-related molecules); ↓ lipid deposition in kidney *; ↓ expression of SREBF1, FAS *, SCD1, PPAR-γ (fatty acid synthesis); ↑ genes expression and protein levels of PPAR-α and CPT-1α (fatty acid β-oxidation); ↓ inflammatory response in kidney (↓ serum content or kidney mRNA levels of TNF-α *, IL-1β *, IL-6 *, MCP-1 *); ↓ NF-κB/ HDAC3 signaling in kidney * group 3–5 compared to group 2 for all tests; * juglanin 7.5–30 mg/kg; juglanin 15–30 mg/kg for all other results	[10]
kidney disease	anti-inflammatory, dyslipidemia improvement; in vitro cellular study; HK2 cells (immortalized proximal tubule epithelial cell line from adult human kidney)	1. normal control 2. cells with palmitate induced pathology 3. cells with palmitate and juglanin (10, 20, 40 μM)	↓ expression or protein levels of SREBF-1 **, FAS **, SCD-1 ***, ACCα *, PPAR-γ ***; ↑ expression or protein levels of PPAR-α **, CPT-1 **, UCP-2 **; ↓ TNF-α ***, IL-1β ***, IL-6 ***, and MCP-1 *** mRNA levels; ↓ p-IKKAα ***, p-IKβα ***, p-NF-κB *** levels; ↑IKβα *** level; inhibition of NF-κB/HDAC3 nuclear translocation *** Group 3 compared to group 2; *** juglanin at 10–40 μM; ** juglanin at 20–40 μM; * juglanin at 40 μM	
adipogenesis	inhibition of adipogenesis in vitro cellular study; 3T3-L1 preadipocytes	1. 3T3-L1 preadipocytes 2–4. 3T3-L1 preadipocytes with juglanin (0.5, 2.5 or 5 μM)	↓ lipid accumulation in differentiated adipocytes (day 8 of the study), the effect reversed in cells treated with AMPK inhibitor; ↓ FABP4 * and GLUT4 *expression in preadipocytes; inhibition of the gene expression of adiponectin * and leptin * during differentiation process; ↓ gene and protein levels of C/EBPα, C/EBPβ and SREBP-1c, no effect on PPAR-α and PPAR-γ; ↑ activation of the SIRT1/AMPK signaling pathway (↑ phosphorylation of AMPKα and SIRT1 mRNA and protein levels) groups 2–4 compared to group 1 for all tests; * juglanin at 5 μM; juglanin at 0.5–5 μM for other results	[84]
obesity	appetite suppression; molecular docking studies	-	favorable binding to 5-HT2C outside of the known agonist binding pocket (juglanin may be positive allosteric modulators of 5-HT2C receptor function)	[85]
diabetes, metabolic syndrome	antidiabetic; in vitro α-glucosidase inhibition	positive control: acarbose (IC_50_ = 1819.72 μM)	weak activity toward α-glucosidase (IC_50_ = 3246.17 μM)	[32]
**Discussed mainly in** Section 5.1**: juglanin and fibrosis treatment; and** Section 5.3**: hepatoprotective potential of juglanin**				
hepatitis caused by fructose	anti-inflammatory, anti-apoptotic, hepatoprotective, hypolipidemic, anti-diabetic; in vivo animal model; male Sprague-Dawley rats; n = 15/group	1. conventional diet 2. fructose-feeding group 3–5. fructose and juglanin (5, 10 or 20 mg/kg p.o.), duration 5 weeks	↓ body weight * and body fat *; ↓ serum endotoxin (LPS) level; ↓ serum glucose level (OGTT, ITT); ↓ TG, TC *, LDL *, and ↑ HDL * levels in serum; ↓ TG and TC levels in liver; ↓ liver inflammation * and liver fibrosis * scores; ↓ level of inflammatory cytokines in serum (TNF-α, IFN-γ, IL-17, IL-10, TGF-β, IL-35, IL-1β, IL-6, IL-18 *); ↓ level cytokines in liver (IL-1β **, IL-6, IL-18); ↓ serum and liver ALT, AST, ALP; ↓ level of TLR4 expressing liver cells, ↓ TLR4 *, MyD88 **, IRAK4 **, IRAK1 **,TRAF6 *, TSK1 ** levels; suppression of MAPK and NF-κB signaling pathway (↓ p-JNK/JNK, p-ERK/ERK, p-p38/p38, c-Jun **, p-IKKα *, p-IκBα **, p-NF-κB/NF-κB and ↑ IKKα *, IκBα * levels in liver); ↓ cytokines expression (TNF-α *, IFN-γ, IL-17, IL-10, TGF-β, IL-35, IL-1β *, IL-6, IL-18 **); ↓ apoptosis (↓ p-JAK2/JAK2 **, p-STAT3/STAT3 *, cyto-c *, caspase-9 *, caspase-3 and ↑ Bcl-xL levels in liver); ↓ cyto-c, caspase-9, caspase-3 expression and ↑ Bcl-xL expression, JAK2 and STAT3 expression not changed groups 3–5 compared to group 2 for all tests; ** juglanin at 20 mg/kg; * juglanin at 10–20 mg/kg; juglanin at 5–20 mg/kg for other results	[86]
**Discussed mainly in** Section 5.3**: hepatoprotective potential of juglanin**				
nonalcoholic fatty liver disease (NAFLD) induced by high-fat diet	anti-inflammatory, hepatoprotective, hypolipidemic, anti-diabetic; in vivo animal model; C57BL/6 mice; n = 6/group	1. low-fat diet control 2. High-fat diet (HFD) control 3–5. HFD and juglanin (5, 10 or 20 mg/kg/day p.o. for 3 weeks), duration 8 weeks	↓ inflammatory lesions and lipid accumulation in mouse liver tissue (histomorphological changes) ^#^; ↓ serum AST *, ALT **, cholesterol **; ↓ fasting serum glucose **, insulin **, HOMA-IR **; ↑ PPAR-α *, FGF21 **, CPT-1a ** mRNA levels, ↑ p-ACC/ACC ** (lipid metabolism); ↓ TNF-α **, IL-6 ^#^ and IL-1β ^#^ levels; ↓ intestinal permeability (↑ expression of tight junction protein ZO-1 **, ↓ FITC-dextran **) groups 3–5 compared to group 2 for all tests; ^#^ lack of statistics; * juglanin at 20 mg/kg; ** juglanin at 10–20 mg/kg; juglanin at 5–20 mg/kg for other results	[87]
non-alcoholic steatohepatitis-like phenotype (hepatic injury) induced by PM_2.5_ (air pollution containing particulate matter less than 2.5 μm)	hepatoprotective, anti-inflammatory, antioxidant; in vivo animal model; wild type male C57BL/6 mice; n = 5–8/group	1. normal control (filtered air) 2. filtered air and juglanin 3. PM_2.5_ control 4. PM_2.5_ air and juglanin (40 mg/kg/day p.o.), duration 24 weeks	body weight not changed; ↓ MBP, ↑ liver weight; ↓ ALT, AST; ↑ SOD activity, ↓ MDA level; ↑ Nrf2 ^#^ and SIKE, ↓ p-TBK1 and p- NF-κB levels; ↑ Nrf2 ^#^ expression; ↑ HO-1, NQO-1 ^#^, GCLC and GCLM ^#^ expression; ↓ IL-1β, IL-6, TNF-α and IFN-β expression group 4 compared to group 3 for all tests (^#^ additionally group 2 compared to group 1)	[11]
hepatic injury	hepatoprotective, anti-inflammatory, antioxidant; in vitro cellular study; human liver cells L02	1. normal cells 2. normal cells and juglanin 3. cells with PM_2.5_ 4. PM_2.5_ (air pollution, particulate matter less than 2.5 μm) and juglanin (40 μM) 5. PM_2.5_ cells and t-BHQ (10 μM, positive control)	↓ Nrf2 nuclear translocation in dose-dependent (5–80 μM) manner (↓ cytoplasm Nrf2 and ↑ nuclear Nrf2—tested for group 2 compared to group 1 only); ↓ oxidative stress (↓ Keap-1 and ↑ HO-1 ^#^ levels;↑ SOD ^#^, CAT ^#^, GPx ^#^ activity; ↓ ROS accumulation), effects comparable to t-BHQ treatment; ↓ inflammation (↑ SIKE ^#^ level, ↓ IL-1β, IL-6, TNF-α, IFN-β expression, ↓ p-TBK1, p-NF-κB levels), effects comparable to t-BHQ treatment group 4 compared to group 3 for all tests (^#^ additionally group 2 compared to group 1)	
**Discussed mainly in** Section 5.4**: Effects of juglanin on UVB-induced skin injury, wound healing and cellular senescence**				
hyperplasia and cell infiltration in the skin caused by UVB radiation	anti-inflammatory, antioxidant; in vivo animal model; female SKH-1 hairless mice; n = 15/group	1. normal control 2. animals irradiated with UVB 3–4. UVB irradiation and juglanin (15 or 30 mg/kg p.o. three times per week), duration 10 weeks	↓ skin injury (↓ skin thickness, MPO activity, transepidermal water loss); ↓ total groove volume *, wrinkle area, wrinkle volume * and the number of wrinkles; ↓ MDA * and TBARS levels (↓ lipid peroxidation); ↑ GSH level, ↑ SOD, CAT and GPx activity; ↓ iNOS and TGF-β1, ↑ Nrf2 expression and levels; ↓ phosphorylation of p38, ERK1/2 and JNK * (↓ MAPK signaling pathway); ↓ inflammatory response (↓ NF-κB phosphorylation; ↓ COX-2, IL-1β *, TNF-α * expression) groups 3–4 compared to group 2 for all tests; * juglanin at 30 mg/kg; juglanin at 15–30 mg/kg for other results	[12]
UVB-induced skin injury	anti-inflammatory, antioxidant; in vitro cellular study; human epidermal cells (HaCaT) exposed to UVB radiation	1. normal control 2. cells treated with UVB 3–4. UVB and juglanin (80 or 160 μM)	↓ ROS generation; ↑ SOD1, SOD2 *, CAT and Nrf2, ↓ TGF-β1 and iNOS expression levels; ↓ p38, ERK1/2 * and JNK * phosphorylation (lack of effect in cells with Nrf2 knock down; synergistic activity with MAPK inhibitors); ↓ NF-κB phosphorylation; ↓ COX-2, IL-1β *, and TNF-α * expression and levels (lack of effect in cells with Nrf2 knock down; synergistic activity with NF-κB inhibitor) groups 3–4 compared to group 2 for all tests; * juglanin at 160 μM; juglanin at 80–160 μM for other results	
wound	wound healing; in vitro scratch-wound healing assay; human dermal fibroblasts (CCD-1064sk)	1. scratch-wound control 2. scratch-wound and juglanin (1 or 5 μM)	promote the migration of fibroblasts (↑ % of wound closure after 16 and 24 h) group 2 compared to group 1	[15]
cellular senescence	inhibition of cellular senescence; in vitro cellular study; human dermal fibroblasts or human umbilical vein endothelial cell	1. normal control 2. cellular senescence induced by Adriamycin 3. adriamycin and juglanin (1, 3, 5 or 10 µg/mL) 4. old cells (replicative senescence) 5. old cells and juglanin (1, 3, 5 or 10 µg/mL) 6. *N*-acetylcysteine (5 mM, positive control) 7. rapamycin (500 nM, positive control)	no effect on senescence-associated β-galactosidase (SA-β-gal) activity observed for human umbilical vein endothelial cell, hence this model was excluded from further studies; effects on human dermal fibroblasts (group 3 compared to group 2): ↓ SA-β-gal activity (at 3–10 µg/mL comparable to both positive controls); ↓ p53 and p21 levels, no effect on pS6 (lack of statistics); ↓ intracellular ROS (tested for 10 µg/mL, comparable to rapamycin control) effects on human dermal fibroblasts (group 5 compared to group 4): ↓ SA-β-gal activity (at 5–10 µg/mL comparable or more effective than both positive controls)	[78]
**Discussed mainly in** Section 5.5**: Juglanin and cardiovascular disorders**				
shear stress-induced endothelial dysfunction (atherosclerosis model)	anti-inflammatory, antioxidant; anti-atherogenic in vitro cellular study; human aortic endothelial cells (HAECs)	1. normal control 2. oscillatory shear stress control 3–4. cells with shear stress and juglanin (2.5 or 5 μM)	↓ NOX-2 protein and mRNA levels, ↓ intracellular ROS levels; ↓ IL-1β and MCP-1 mRNA and protein expression, ↓ HMGB1 secretion (↑ HMGB1 release in normal cells at 25–50 μM dose); ↑ eNOS protein and mRNA levels, ↑ NO concentration; ↓ attachment of TPH-1 monocytes to endothelial cells, ↓ VCAM-1 and E-selectin mRNA and protein expression; ↑ atheroprotection (KLF-2 mRNA and protein expression) groups 3–4 compared to group 2 for all tests	[79]
acute myocardial infarction (AMI) induced by ligation of the left anterior descending artery	anti-inflammatory, anti-apoptotic in vivo animal model; male Sprague–Dawley mice; n = 8/group	1. normal control 2. mice with AMI 3–4. mice with AMI and juglanin (10 or 30 mg/kg/day), duration 14 days	improved the morphological changes in myocardial tissue (↓ loose, edematous and necrotic cardiomyocytes, ↓ inflammatory cell infiltration); ↓ LDH and CK-MB; ↓ inflammatory response (↓ IL-6, IL-1β, TNF-α, and ↑ IL-10 levels in serum); ↓ apoptosis (↓ TUNEL-positive cells, ↓ caspase-3 and caspase-9 levels); MAPK signaling pathway regulation (↓ phosphorylation of p-38, p-ERK, p-JNK) groups 3–4 compared to group 2 for all tests	[7]
cerebral ischemia	anti-ischemic; in vivo animal model; C57/BL6 mice; n = unknown	1. normal control 2. normal mice with juglanin 3. mice with middle cerebral artery occlusion (MCAO) 4. mice with MCAO and juglanin (20 mg/kg p.o. for 3 weeks before the MCAO)	↓ infarct volume by about half; ↓ neurological score, ↓ blood-brain barrier permeability; ↓ VEGF and VEGFR2 mRNA and protein levels (inhibition of MCAO-induced angiogenesis); ↑ expression of the tight junction proteins (occludin and ZO-1) ^#^ group 4 compared to group 3 for all tests (^#^ additionally group 2 compared to group 1)	[88]
	anti-ischemic; in vitro cellular study; bEnd.3 human brain microvascular endothelial cells exposed to oxygen–glucose deprivation/reperfusion (OGD/R)	1. normal control 2. cells with OGD/R 3–4. cells with OGD/R and juglanin (2.5 or 5 μM)	↑ cells viability, ↓ release of lactate dehydrogenase (↓ cytotoxicity caused by OGD/R); ↓ brain endothelial permeability; ↑ occludin and ZO-1 protein levels; ↓ VEGF * and VEGFR2 * protein levels (the addition of VEGF-A to the experiment abolished the effects of juglanin on brain endothelial permeability) groups 3–4 compared to group 2 for all tests; * juglanin at 5 μM; juglanin at 2.5–5 μM for other results	
oxidative modifications of biomolecules	antioxidant; ex vivo model on human plasma	1. normal control 2. human plasma exposed to ONOO^–^ in vitro 3–5. ONOO^–^ treated plasma and juglanin (1, 5 or 50 μg/mL) 6. ONOO^–^ treated plasma and ascorbic acid, AA (1, 5 or 50 μg/mL)	↓ nitration of protein tyrosine residues (↓ 3-NT levels); ↓ levels of TBARS; ↑ NEAC * of plasma; effects comparable to AA at 1–5 μg/mL, AA activity at 50 μg/mL higher groups 3–5 compared to group 2 for all tests; * 1–5 μg/mL; juglanin at 1–50 μg/mL for other results	[89]
	antioxidant; ex vivo model on human fibrinogen	1. normal control 2. fibrinogen exposed to ONOO^–^ in vitro 3–5. ONOO^–^ treated fibrinogen and juglanin (1, 5 or 50 μg/mL) 6. ONOO^–^ treated fibrinogen and ascorbic acid (1, 5 or 50 μg/mL)	↓ high molecular weight aggregates; no effects on Aα, Bβ and γ bands; ↓ 3-NT level; ↓ tryptophan residue oxidation *; effects comparable to AA at 1–5 μg/mL, AA activity at 50 μg/mL higher groups 3–5 compared to group 2 for all tests; * juglanin at 50 μg/mL; juglanin at 1–50 μg/mL for other results	
oxidative stress-related disorders	antioxidant; in vitro studies	positive control: ascorbic acid (AA, 5.68 µmol/mg)	no effect on O_2_^•−^, NO^•^, ONOO^−^; low scavenging potential toward H_2_O_2_ (SC_50_ = 0.64 µmol AA equivalents/mg); high scavenging potential toward HO^•^ (SC_50_ = 6.35 µmol AA equivalents/mg) and HClO (IC_50_ = 8.56 µmol AA equivalents/mg)	[90]
		positive control: curcumin (SC_50_ = 27.80 μM)	DPPH scavenging: IC_50_ = 89.91 μM	[3]
		positive control: ascorbic acid (SC_50_ = 1.76 μg/mL for DPPH assay)	DPPH scavenging: IC_50_ = 0.16 μg/mL; β-carotene-linoleic acid assay: lower activity than ascorbic acid	[52]
		positive control: L-penicillamine (SC_50_ = 4.62–6.90 μM)	ONOO^−^ scavenging: SC_50_ = 22.3 μM	[24,39]
		positive controls: ascorbic acid (DPPH, IC_50_ = 11.5 μM), trolox (ROS inhibition 73.6%), penicillamine (ONOO^−^ inhibition, IC_50_ = 3.2 μM)	DPPH scavenging: IC_50_ = 100.4 μM; Total ROS inhibition (in vitro on rat kidney homogenates): 36.8% ONOO^−^ inhibition: IC_50_ = 10.5 μM	[43]
		positive control: Trolox	peroxyl radical-scavenging activity (ORAC): about 12 µM Trolox equivalents; hydroxyl radical-scavenging activity (ORAC): about 8 µM Trolox equivalents; metal chelating (Cu^2+^) and reducing (copper (I) ions) potential	[31]
		positive control: Trolox	peroxyl radical-scavenging activity (ORAC): 0.62 Trolox equivalents	[21]
blood coagulation-related disorders	anti-coagulant; ex vivo study on platelet aggregation in human blood; in silico molecular docking and in vitro study of thrombin activity	1. control platelet-rich human plasma stimulated with adenosine diphosphate (ADP) or collagen 2. stimulated plasma and juglanin (5 or 50 µg/mL) 3. stimulated plasma and indomethacin (positive control, 5 µg/mL)	weak effect on platelet aggregation after ADP stimulation * (↓ aggregation by <15%, for indomethacin ↓ by 23%); no effect on platelet aggregation after collagen stimulation (for indomethacin ↓ by 86%); no effect on thrombin activity group 2 compared to group 1; * juglanin at 50 μg/mL; juglanin at 1–50 μg/mL for other results	[4]
blood coagulation-related disorders	pro-coagulant; ex vivo study on platelet aggregation in New Zealand white rabbit blood	1. control blood sample 2. blood sample with juglanin (2 mg/mL) 3. blood sample with breviscapine (13.3 mg/mL)	no effect on APTT and PT times; ↓ TT time (weaker effect than breviscapine); ↑ fibrinogen content (comparable effect to breviscapine) group 2 compared to group 1	[48]
inflammation-related disorders	anti-inflammatory; in vitro cellular study; Raw264.7 cells	1. normal control 2. LPS-stimulated control 3. LPS and juglanin (5 μg/mL)	no significant effect on NO release group 3 compared to group 2	[22]
**Discussed mainly in** Section 5.6**: Juglanin and central nervous system disorders**				
cognitive impairment caused by doxorubicin	antioxidant, anti-inflammatory, anti-apoptotic; in vivo animal model; male rats; n = 6/group	1. normal control 2. doxorubicin control 3. normal rats and juglanin 4. doxorubicin and juglanin (30 mg/kg/day p.o.), duration 4 weeks	↑ body weight; memory and cognitive function improved (Y-maze test: ↑ % of spontaneous alternation, total arm entries not changed; Morris water maze test: ↓ the latency of escapes; space exploration test: ↑ the time spent in the target quadrant and number of crossings); depression-like behavior improved (forced swimming test: ↓ immobility time, ↑ swimming and climbing time); ↓ inflammatory mediators levels in the brain (↓ TNF-α, IL-1β, IL-6 and NF-kB); ↓ oxidative stress in the brain (↓ MDA level, ↑ SOD, CAT, GSH); alleviated histopathological alteration in the brain (pyknosis, degenerated and swollen neurons and congested blood vessels); ↓ activity of AchE and caspase-3 group 4 compared to group 2 for all tests; no effect of juglanin on healthy rats	[83]
chronic unpredictable mild stress (CUMS)	improvement of anxiety/depression-like behaviors, neuroprotective, anti-inflammatory; in vivo animal model; male C57BL/6N mice; n = 4–8/group	1. normal control 2. normal mice and juglanin 3. CUMS control 4. CUMS and juglanin (30 mg/kg/day p.o), duration 8 weeks	↑ sucrose consumption, ↓ immobility and transfer latency time, ↑ time spend in the center zone, target quadrant and the frequency of appearance in the target quadrant (behavior tests: tail suspension test; open-field test; Morris water maze test); effects on depression-related hormones (↑ 5-HTP serum level, ↑ 5-HT, DA and GABA hippocampal levels, ↓ CORT serum and hippocampal levels, ↓ ACTH serum level, ↓ glutamate hippocampal level); ↓ neuronal damage in hippocampal section; ↑ expression of p-AKT, PI3K, and BDNF in hippocampus, ↓ cleaved caspase-3 and cleaved PARP protein expression, ↑ Bcl-2 protein and gene expression, ↓ Bax gene expression; ↓ the activation of microglial cells and astrocytes (↓ CD11b and GFAP levels); ↓ neuroinflammation (↓ TNF-ɑ, IL-1β, IL-6, MCP-1 serum and hippocampal levels/expression, ↓ iNOS and COX-2 gene expression, ↓ p-IKKɑ, p-IκBɑ and p-NF-κB protein expression) ↑ AMPK activation (↑ p-AMPK) group 4 compared to group 3 for all tests, no effect of juglanin on healthy mice	[6]
neuroinflammation	neuroprotective, anti-apoptotic; in vitro cellular study; mouse hippocampal cell line HT-22	1. normal control 2. normal cells and juglanin 3. LPS control 4. LPS and juglanin (40 μM)	↓ apoptotic cell death (↓ TUNEL-positive cells); ↓ Bax and ↑ Bcl-2 gene expression; ↑ PI3K, p-AKT, Bcl-2 protein expression; ↓ cleaved caspase-3 and cleaved PARP protein expression; ↑ p-AMPK protein expression; the observed effects were reversed in cells with silenced AMPK group 4 compared to group 3 for all tests, no effect of juglanin on normal cells	
	neuroprotective, anti-inflammatory; in vitro cellular study; an immortalized murine microglial cell line BV2	1. normal control 2. normal cells and juglanin 3. LPS control 4. LPS and juglanin (40 μM)	↓ microglial cell activation (↓ CD11b expression); ↓ nuclear NF-κB expression; ↓ p-IKKɑ, p-IκBɑ, p-NF-κB and NF-κB protein expression; ↓ TNF-ɑ, IL-1β, IL- 6, IL-18, MCP-1, iNOS and COX-2 gene expression; ↓ TNF-ɑ, IL-1β level; ↑ p-AMPK protein expression; the observed effects were reversed in cells with silenced AMPK group 4 compared to group 3 for all tests, no effect of juglanin on normal cells	
neuroinflammation in Parkinson’s disease	anti-inflammatory, neuroprotective; in vivo animal model; male C57BL6 mice; n = 20/group	1. normal control 2. LPS control 3–5. LPS and juglanin 10, 20 or 30 mg/kg p.o., five times per week), duration 13 days	attenuation of the cognitive dysfunction (↓ time to find hidden platform; ↑ number of platform crossings and time spend in target quadrant in Morris water maze test); ↑ SYP, PSD-95 and SNAP-25 *, ↓ Aβ and p-Tau expression and proteins levels in hippocampus; ↓ α-synuclein and ↑ TH expression and levels in hippocampus and/or Substantia Nigra; ↓ expression and levels of proinflammatory cytokines (IL-1β, IL-18, TNF-α) and COX-2; ↓ TLR4 **, MyD88 ^#^, CD14 ^#^, p-IKKα, p-IκBα and p-NF-κB levels; ↓ astrocytes and microglia activity, protective activity against neurons damages (↓ GFAP positive cells, ↓ Iba1 expression) groups 3–5 compared to group 2 for all tests; ** juglanin at 30 mg/kg; * juglanin at 20–30 mg/kg; ^#^ lack of statistics; juglanin at 10–30 mg/kg for other results	[76]
neuroinflammation	anti-inflammatory, neuroprotective; in vitro cellular study; mice primary astrocytes	1. normal control 2. LPS control 3–5. cells with LPS and juglanin (40, 80 or 160 μM)	↓ TLR4/MyD88/CD14 ^#^, p-IKKα ^#^, p-IκBα ^#^ and p-NF-κB ^#^; ↓ GFAP ^#^, BDNF ^#^ and ↑ TH ^#^ levels; ↓ GFAP and p-NF-κB activity; ↑ SYP ^#^, PSD-95 ^#^ and SNAP-25 ^#^ levels; ↓ Aβ ^#^ and p-Tau ^#^ levels; ↓ levels of proinflammatory cytokines (IL-1β, IL-18 *, TNF-α) and COX-2 * groups 3–5 compared to group 2 for all tests; * juglanin at 80–160 μM; ^#^ lack of statistics; juglanin at 40–160 μM for other results	
**Discussed mainly in** Section 5.7**: Juglanin and skeletal system disorders**				
model of bone loss induced by ovariectomy	inhibition of osteoclastogenesis; anti-osteoporotic in vivo animal model; female C57BL6 mice; n = 10/group	1. normal control 2. ovariectomized mice 3. ovariectomized mice and juglanin (10 mg/kg intraperitoneal injection every two days for 8 weeks)	↓ bone loss (↓ number of osteoclasts, ↑ bone volume/tissue volume, ↑ trabecular number, ↓ trabecular separation, trabecular thickness not changed); group 3 compared to group 2	[82]
osteoclastogenesis	inhibition of osteoclastogenesis, anti-osteoporotic in vitro cellular study; bone marrow mononuclear cells BMMs (extract from C57BL/6 mice); macrophage call line RAW264.7; bone marrow stromal cells (BMSCs)	1. normal control 2. cells with RANKL (differentiation of osteoclasts) 3. cells with RANKL and juglanin (20, 40, 80 μmol/L)	↓ number of osteoclasts **; ↓ osteoclasts function (↓ the size of F-actine ring and number of nuclei **, ↓ the resorptive function of osteoclasts **); ↓ RANKL-induced osteoclast formation at early stage; ↓ osteoclastogenic gene expression (of c-Fos **, TRAcP **, MMP-9 ** and CTSK **); ↓ NFATc1 ** transcriptional activity and V-ATP-ase-d2 expression; ↓ NF-κB * transcriptional activity, phosphorylation of IκBα, p65 and p50, and p65 nuclear translocation; no inhibitory or promotive effect on BMSCs differentiation (alkaline phosphatase and alizarin red staining assays) group 3 compared to group 2; ** juglanin at 20–80 μmol/L; * juglanin at 40–80 μmol/L; juglanin at 80 μmol/L for other results	
osteoarthritis	anti-inflammatory; in vitro cellular study; primary human osteoarthritis chondrocyte culture	1. normal control 2. inflammation induced by IL-1β 3–5. cells with IL-1β and juglanin (10, 20 or 40 μM)	↑ cell viability; ↓ NO and PGE2 production, ↓ iNOS and COX-2 mRNA and protein expression; ↓ TNF-α, IL-6, MMP-1, MMP-3, MMP-13 levels; ↓ expression of ADAMTS-4 and ADAMTS-5; ↓ p-p65 and ↑ IκBα expression (NF–κB pathway) Groups 3–5 compared to group 2 for all tests	[13]
arthritis	anti-inflammatory, antioxidant; in vitro cellular study; chondrogenic ATDC5 cells	1. normal control 2. cells with LPS 3–4. cells with LPS and juglanin (2.5 or 5 μM)	↓ oxidative stress (↓ ROS level, ↑ SOD activity, ↓ NOX-4 expression); ↓ activation of the NLRP3 inflammasome complex (↓ TxNIP gene and protein levels, ↓ NLRP3, ACS, P10 levels); ↓ IL-1β and IL-18 secretion; ↑ SIRT1 gene and protein levels (silencing of SIRT1 abolished the effects of juglanin against activation of NLRP3 inflammasome) groups 3–4 compared to group 2 for all tests	[91]
adjuvant-induced arthritis (AIA) by Freund’s complete	anti-inflammatory, antioxidant, hepatoprotective; in vivo animal model; female Wistar rats; n = 12/group	1. normal control 2. AIA control 3. AIA and leflunomide (10 mg/kg) 4–6. AIA and juglanin (10, 20, 40 mg/kg p.o), duration 16 days	↓ levels of hepatic TNF-α, IL-6, TNF-α, TGF-β, COX-2 and iNOS mRNA; ↓ hepatic NF-κB, IκBα, ADAMTS-4, and ADAMTS-5 proteins expression; ↓ hepatic NO and MDA levels; ↑ hepatic GSH and SOD levels; ↓ rheumatoid factor ↓ level of TG, LDL-C, TC, and VLDL-C; ↑ levels of HDL-C; ↓ serum AST, ALT, ALP, and CRP levels; ↑ level of albumin in serum; ↑ levels of RBC and Hb; ↓ level of WBC, platelets and ESR ↑ body and liver weight; ↑ paw withdrawal latency and threshold; ↓ joint diameter and paw volume attenuation of inflammatory cells infiltration, and cartilage and synovial destruction (histopathological analysis) groups 5–6 compared to group 2 for all tests (dose of juglanin 20–40 mg/kg); effects comparable to after leflunomide (10 mg/kg) treatment	[92]
**Discussed mainly in** Section 5.8**: Juglanin and anti-cancer potential**				
skin cancer induced by UVB	anti-inflammatory, pro-apoptotic, anti-cancer; in vivo animal model; hairless SKH-1 mice; n = 10/group	1. normal control 2. UVB irradiation group (twice a week) 3–4. UVB irradiation and juglanin (10 or 20 mg/kg p.o., twice per week), duration 10 weeks	↓ skin vasculate *, epidermal hyperplasia and inflammatory cell infiltration *; ↓ KI67 * expression (marker of proliferation); ↓ p-p38/p38, p-JNK/JNK *, PI3K/GAPDH *, p-AKT/AKT *, p-mTOR/mTOR *; ↓ inflammation (↓ IL-1β *, IL-18 *, TNF-α *, p-NF-κB/NF-κB *); ↓ expression of cycline D1 *, CDK1 *, PCNA *, ↑ expression of p53 *, p27 *, p21 *; ↑ caspase-3 *, caspase-8 *, and PARP-1 * levels (enhanced apoptotic cell death) * juglanin 10–20 mg/kg groups vs. UVB control group; juglanin at 20 mg/kg group vs. UVB control for other results	[81]
	anti-inflammatory, pro-apoptotic; in vitro cellular study; B16F10 murine melanoma cells (tumor line)	1. control cells 2. cells treated with UVB 3. cells treated with UVB and juglanin (2.5–20 μM)	↓ cells proliferation *^#^ (MTS cell proliferation colorimetric assay–mitochondrial activity); ↑ apoptotic cells *; ↑ caspase-3 *, caspase-8 *, and PARP * levels; Inhibition of cells proliferation (↓ levels of p-p38 *, p-JNK/JNK *,p-NF-κB *; ↑ levels of p53 *^#^, p27 *^#^, p21 *^#^) ^#^ group 3 compared to group 2; *Group 3 compared to group 1	
breast cancer (tumor-transplanted mouse model)	anti-cancer, pro-apoptotic; in vivo animal model; male BALB/c-nude mice; n = 10/group	1. mice with breast cancer xenograft 2–3. mice with breast cancer and juglanin 5 or 10 mg/kg/day p.o.), duration 7 days	body weight not changed; ↓ tumor volume; ↑ level of cleaved caspase-9 and caspase-3, LC3BI, LC3BII and phosphorylated JNK; ↑ dead cells and expression level of cleaved caspase-9 groups 2 and 3 compared to group 2 for all tests	[77]
breast cancer	anti-cancer, pro-apoptotic; in vitro cellular study; human breast cancer cell lines MCF-7, SKBR3 (for all tests), MDA-MB-231 and BT474 (only for the viability test)	1. breast cancer cells 2. cells with juglanin (2.5, 5, 10 μM for all tests, and up to 40 μM for the viability test)	↓ cell proliferation (↓ cell viability at 10–40 μM for all cell lines; MTS cell proliferation colorimetric assay–mitochondrial activity); ↓ the number of cell colonies, induction of G2/M phase arrest (↑ phosphorylation of Chk2, Cdc25C, Cdc2, ↑ p27, ↓ cyclin D); ↑ apoptotic cells level (↓ Bcl-2 ^#^ expression, ↑ Bad ^#^ and Bax ^#^ level, ↑ expression of cleaved casapse-9 ^#^, casapse-8 ^#^ and caspase-3 ^#^); ↑ autophagy (↑ production of ROS and ↑ activation of JNK ^#^) group 2 compared to group 1 for all tests; ^#^ lack of statistics; juglanin at 2.5–10 μM for other results	
xenograft tumor model (lung cancer cells A549 transplantation)	anti-cancer, pro-apoptotic, autophagy induction in vivo animal model; athymic, nude mice; n = 10/group	1. mice with cancer 2–4. sick mice with juglanin (10, 20 or 30 mg/kg/day p.o.), duration 28 days	body weight and liver mass not changed; ↓ tumor volume and weight (after 28 days); ↓ tumor KI-67 positive cells *, ↑ autophagy and apoptosis levels; ↑ cleavage of Caspase-3 * and PARP *; ↓ Bcl-2 * and ↑ Bax * levels; ↑ p53, TRAIL, DR4, DR5 and FADD mRNA expression; ↑ IκBα, ↓ p-IκBα * and p-NF-κB * protein levels; ↓ PI3K*, AKT*, and ERK1/2 phosphorylation, ↑ p-p38; ↑ LC3 I/II, ATG-7, Beclin-1 and PIK3C3 expression groups 2–4 compared to group 1 for all tests; * juglanin at 20–30 mg/kg; juglanin at 10–30 mg/kg for other results	[8]
lung cancer	anti-cancer, pro-oxidant, pro-apoptotic, autophagy induction in vitro cellular study; lung cancer cell lines A549, H1975 (and HCC827 only for viability test)	1. cancer cells 2. A549 or H1975 cells and juglanin (20, 30, 40 μM)	↓ cells viability (10–80 μM for A549 cells, 5–80 μM for H1975 and HCC827 cells; MTT assay–mitochondrial activity); chromatin condensation *; ↑ apoptotic cells *; ↑ cleavage of caspase-8 ***, caspase-9 ***/**, caspase-3 ***, and PARP ***/**; ↓ Bcl-2 ***/** and Bcl-xL **/*, ↑ Bax ** and Bad *** levels; (effects on caspase-9, caspase-3 and call viability reversed with caspase inhibitors); ↑ TRAIL ***/**, DR4 ***, DR5 *** and FADD *** mRNA levels, ↑ c-Abl ***, p73 ***/** and p53 *** protein levels (effects reversed with the p53 inhibitor, resulted in apoptotic cells ↓); regulation of NF-κB, PI3K/AKT and MAPKs signaling pathways: ↓ NF-κB ***/**, p-IκBα **, p-PI3K **/***, p-AKT ***/**, p-ERK1/2 *** protein levels, ↑ IκBα */**, p-p38 ***, p-JNK *** and p-cJun *** protein levels (apoptosis enhanced with the use of PI3K/AKT inhibitor or p38 activator or ERK1/2 inhibitor or JNK activator); ↑ ROS production * (the effects on cell viability and apoptosis reversed with the use of ROS scavenger); autophagy induction: ↑ LC3 ***, ATG-7 ***, ATG-3 **, Beclin-1 **/***, PIK3C3 ***, and AMBRA1 **/*** levels group 2 compared to group 1 for all tests; * juglanin at 40 μM, ** juglanin at 30–40 μM, *** juglanin at 20–40 μM (active dose for A549/H1975 cells)	
cancer	cytotoxic; in vitro cellular study on different human cancer cell lines	positive control: doxorubicin (IC_50_ = 0.001–0.02 µM depending on cell lines)	no toxicity observed based on sulforhodamine B assay–protein content, IC_50_ > 100 µM on A549 (non-small cell lung adenocarcinoma), SK-OV-3 (ovarian cancer cells), SK-MEL-2 (skin melanoma cells), HCT-15 (colon cancer cells)—no effects on cells proliferation	[25]
cancer	cytotoxic; in vitro cellular study on Ehrlich ascitis carcinoma cells	cells treated with juglanin 25, 50 or 100 μg/mL	↓ cells viability (>90% inhibition)	[30]
**Discussed mainly in** Section 5.9**: Juglanin and antifungal, antiviral and antiparasitic potential**				
fungal infections	antifungal; in vitro study	positive control: fluconazole	*Candida albicans:* for ATCC 90028, 03, 48, 111, 181 strains MIC > 128 μg/mL; for the 02 strain MIC = 128 μg/mL (fluconazole = 2 μg/mL); for the 138 strain MIC = 16 μg/mL (fluconazole = 8 μg/mL); *Candida parapsilosis*: for the ATCC 22019 strain MIC = 128 μg/mL; for the 11 strain MIC = 64 μg/mL (fluconazole = 1 μg/mL)	[33]
malaria	antiplasmodial, cytotoxic; in vitro study on *Plasmodium falciparum* strain 3D7 and HeLa (human cervix adenocarcinoma) cells	positive controls: chloroquine (IC_50_ = 0.014 µM) for *Plasmodium* 3D7; emetin (IC_50_ = 0.04 µM) for HeLa (human cervix adenocarcinoma) cells	↓ parasite viability (about 16.5% viability at 120 µM and about 73.6% viability at 24 µM); ↓ HeLa cells viability (about 2.8% viability at 120 µM and about 71.8% viability at 24 µM)	[35]
virus infections (e.g., SARS coronavirus)	anti-viral; in vitro cellular study on Xenopus oocyte cells with a heterologously expressed 3a protein	-	IC_50_ of 3a-protein channel = 2.3 µM; at 10 and 20 µM near-complete and complete inhibition—juglanin activity was the highest from all tested flavonoids, potential to ↓ virus release	[93]
SARS-CoV-2	anti-viral; computational studies: QSAR analysis and molecular docking studies	positive control: remdesivir	potential inhibition of the 3CL^PRO^ of SARS-CoV-2 (the best binding score from 23 tested flavonoids, better than positive control), potential to ↓ viral replication complex and transcription	[94]
	anti-viral; computational studies: molecular docking studies	positive controls e.g., ritonavir, lopinavir, trametinib, selumetinib	good binding affinity to 3CL^PRO^ (better than ritonavir and lopinavir), PL^PRO^ (better than lopinavir), ABL1 (better than selumetinib, worse than trametinib), and TMPRSS2; no effect on RdRp, non-structural proteins (nsp10, nsp14, nsp15, nsp16), hACE2R, S protein, NFAT	[95]
influenza	anti-viral; molecular docking studies	-	good binding affinity to the surface glycoproteins of influenza virus (hemagglutinin and neuraminidase)	[96]
dengue disease	anti-viral; computational studies: QSAR analysis and molecular docking studies	positive controls: octyl beta-D-glucopyranoside and 2-acetamido-2-deoxy-beta-D-glucopyranose	potential inhibition of dengue virus 2 (DENV2) envelope protein (the lowest atomic contact energy (ACE) score in the molecular docking studies of 54 flavonoids, both the docking score and ACE score better than the positive controls, but unstable conformation based on molecular dynamic simulation)	[97]
**Discussed mainly in** Section 5.10**: other effects**				
-	enzyme inhibitory potential; computational studies: structural activity relationship analysis using density functional theory	-	prediction of affinity toward enzymes: the best for lyase (26.65%), next family A-G protein coupled receptor (19.96%), next hydrolase/kinase/oxidoreductase/phosphodiesterase (6.69%)	[98]
diabetes	improvement of diabetes complications; in vitro rat lens aldose reductase inhibition	73 flavonoids tested, no positive control	64.8% inhibition at 10 µM; 6.3% inhibition at 1µM	[99]
hearing loss	cell-protective; in vivo animal model; wild-type larvae zebrafish (*Danio rerio*); n = unknown	1. normal control 2. neomycin-induced hair cell damage 3. neomycin and juglanin (1 µM for 8h)	↑ number of otic hair cells (significant but moderate effect) group 3 compared to group 2 for all tests	[36]
	cell-protective; in vitro cellular study; mouse auditory cell line HEI-OC1	1. normal control 2. neomycin-induced cell damage 3. neomycin and juglanin (1 µM)	↑ cell viability (from about 63% to about 95%) group 3 compared to group 2 for all tests	

↑ increase; ↓ decrease. Abbreviations: 2,2-Diphenyl-1-picrylhydrazyl (DPPH); 3-chymotrypsin-like protease (3CL^PRO^); 5-hydroxytryptamine (5-HT); 5-hydroxytryptamine receptor 2C (5-HT2C); 5-HTP5-hydroxytryptophan (5-HTP); Abelson murine leukemia viral oncogene homolog 1 (ABL1); acetylcholinesterase (AchE); acetyl-CoA carboxylase α (ACCα); adjuvant-induced arthritis (AIA); activated partial thromboplastin time (APTT); signal transducer and activator of transcription 3 (STAT3); acute coronary syndrome (ACS); acute myocardial infarction (AMI); adenosine diphosphate (ADP); adrenocorticotropic hormone (ACTH); air pollution containing particulate matter (PM); alanine aminotransferase (ALT); alkaline phosphatase (ALP); AMP-activated protein kinase (AMPK); antigen Kiel 67 (KI67); area under the ROC Curve (AUC); aspartate aminotransferase (AST); autophagy protein 7, 3 (ATG-7, ATG-3); autophagy-related gene-related (ATG) protein (AMBRA1); B-cell lymphoma 2 (Bcl-2); B-cell lymphoma-extra large (Bcl-xL); bcl-2-like protein 4 (Bax); beta-amyloid (Aβ); blood urea nitrogen (BUN); bronchoalveolar lavage fluid (BALF); bronchoalveolar lavages (BAL); calcineurin-nuclear factor of activated T-cells (NFAT); carnitine-palmitoyl transferase-1α (CPT-1α); catalase (CAT); CCAAT-enhancer-binding protein α/β (C/EBP α/β); cell division cycle 2 (Cdc2); cell division cycle 25C (Cdc25C); checkpoint kinase 2 (CHK2); checkpoint kinase 2 (Chk2); chronic kidney disease (CKD); c-Jun N-terminal kinase (JNK); cluster of differentiation 14 (CD14); collagen type I alpha 1 (COL1A1); collagen type I alpha 2 (COL1A2); connective tissue growth factor (CTGF); corticosterone (CORT); creatine kinase myocardial band (CK-MB); C-X-C motif chemokine ligand 1 (CXCL1); cyclooxygenase 2 (COX-2); cyclin-dependent kinase 1 (CD-K1); cysteine cathepsin K (CTSK); death receptor 4 (DR4); death receptor 5 (DR5); dengue virus 2 (DENV2); disintegrin and metalloproteinase with thrombospondin motifs-4 and -5 (ADAMTS-4, ADAMTS-5); dopamine (DA); endothelial nitric oxide synthase (eNOS); enhanced neurotrophic factor (BDNF); extracellular signal-regulated kinase (ERK); factor matrix metalloproteinase 1 (MMP-1); factor matrix metalloproteinase 13 (MMP-13); factor matrix metalloproteinase 3 (MMP-3); factor matrix metalloproteinase 9 (MMP-9); FAS-associated death domain protein (FADD); fatty acid synthase (FAS); fatty acid-binding protein 4 (FABP4); fibroblast growth factor 21 (FGF21); fibronectin 1 (FN 1); fluorescein isothiocyanate-dextran (FITC-Dextran); gamma-aminobutyric acid (GABA); glial fibrillary acidic protein (GFAP); glucose tolerance tests (GTT); glucose transporter 4 (GLUT4); glutamate-cysteine ligase subunits (GCLC, GCLM); glutathione (GSH); glutathione peroxides (GPx); high fat diet (HFD); high mobility group box 1 (HMGB1); high-density lipoprotein (HDL); histone deacetylase 3 (HDAC3); homeostatic model assessment for insulin resistance (HOMA-IR); human angiotensin converting enzyme 2 receptor (hACE2R); human mannan-binding protein (MBP); idiopathic pulmonary fibrosis (IPF); immunoglobulin A (IgA); immunoglobulin E (IgE); inducible nitric oxide synthase (iNOS); inhibitor κB-α (IκBα); insulin tolerance test (ITT); interferon γ (IFN-γ); interleukin 10 (IL-10); interleukin 17 (IL-17); interleukin 18 (IL-18); interleukin 1β (IL-1β); interleukin 35 (IL-35); interleukin 4 (IL-4); interleukin 6 (IL-6); interleukin-1 receptor-associated kinase 1 (IRAK1); interleukin-1 receptor-associated kinase 4 (IRAK4); ionized calcium-binding adaptor molecule 1 (Iba1); IκB kinaseα (IKKα); Janus Kinase 2 gene (JAK2); kidney injury molecule-1 (KIM-1); kinase inhibitor 67 (KI67); kruppel-like factor 2 (KLF-2); lactate dehydrogenase (LDH); L-ascorbic acid (AA); lipopolysaccharide (LPS); low-density lipoprotein (LDL); low-density lipoprotein cholesterol (LDLC); macrophage colony stimulating factor (MCS-F); malondialdehyde (MDA); mammalian target of rapamycin (mTOR); microtubule-associated protein 1-light chain 3 beta (LC3BI); microtubule-associated protein 2-light chain 3 beta (LC3BII); microtubule-associated protein light chain 3 (LC3); middle cerebral artery occlusion (MCAO); minimal inhibitory concentration (MIC); mitogen-activated protein kinase (MAPK); monocyte chemoattractant protein 1 (MCP-1); myeloid differentiation primary response 88 (MyD88); myeloperoxidase (MPO); NAD(P)H quinone dehydrogenase 1 (NQO-1); NADPH oxidase 4 (NOX-4); nitrogen oxide (NO); nonalcoholic fatty liver disease (NAFLD); nonalcoholic steatohepatitis (NASH); non-structural proteins 10, 14, 15, 16 (nsp10, nsp14, nsp15, nsp16); nuclear factor erythroid 2-related factor 2 (Nrf2); nuclear factor-κB (NF-κB); nuclear factor of kappa light polypeptide gene enhancer in B-cells inhibitor alpha (IκBα); nucleotide-binding domain, leucine-rich–containing family, pyrin domain–containing-3 (NLRP3); oral glucose tolerance test (OGTT); oxygenase 1 (HO-1); oxygen–glucose deprivation/reperfusion (ODG/R); palmitate (PA); papain-like protease (PL^PRO^); periodic acid-Schiff (PAS); peroxisome proliferator-activated receptor α/γ (PPAR-α/γ); peroxyl radical-scavenging activity (ORAC); phosphoinositide 3-kinase (PI3K); phosphorylated ACC (p-ACC); phosphorylated signal transducer and activator of transcription 3 (p-STAT3); phosphorylated AMP-activated protein kinase (p-AMPK); phosphorylated c-Jun N-terminal kinase (p-JNK); phosphorylated extracellular signal-regulated kinase (p-ERK); phosphorylated inhibitor κB-α (p-IκBα); phosphorylated IκB kinase α (p- IKKα); phosphorylated Janus Kinase 2 gene (p-JAK2); phosphorylated microtubule-associated protein (p-Tau); phosphorylated nuclear factor-κB (p-NF-κB); phosphorylated protein kinase B (p-AKT); phosphorylated TANK-binding kinase 1 (p-TBK1); phosphorylated transcription factor Jun (p-c-Jun); plasma non-enzymatic antioxidant capacity (NEAC); poly-ADP-ribose polymerase (PARP); postsynaptic density protein 95 (PSD-95); proliferating cell nuclear antigen (PCNA); prostaglandin E2 (PGE2); protein kinase B (AKT); prothrombin time (PT); quantitative structure-activity relationship (QSAR); reactive oxygen species (ROS); receptor activator for nuclear factor κB ligand (RANKL); RNA-dependent RNA polymerase (RdRp); senescence-associated β-galactosidase activity (SA-β-gal); sirtuin 1 (SIRT1); spike glycoprotein (S protein); spontaneous mutation (SD); stearoyl-CoA desaturase 1 (SCD1); sterol regulatory element-binding factor 1 (SREBF1); sterol regulatory-element binding proteins 1c (SREBP-1c); stimulator of interferon genes (STING); superoxide dismutase (SOD); suppressor of IKKepsilon (SIKE); synaptophysin (SYP); synaptosomal-associated protein of 25 kDa (SNAP-25); TANK-binding kinase 1 (TBK1); tartrate-resistant acid phosphatase (TRAcP); toll-like receptor 4 (TLR4); terminal deoxynucleotidyl transferase dUTP nick-end labeling (TUNEL); tert-butylohydroquinone (t-BHQ); chronic unpredictable mild stress (CUMS); the house ear institute-organ of corti 1 (HEI-OC1); lowest atomic contact energy (ACE); thiobarbituric acid reactive substance (TBARS); thioredoxin-interacting protein (TxNIP); thrombin time (TT); tight skin 1 (TSK1); tissue inhibitor of metalloproteinase 1 (TIMP-1); TNF-related apoptosis-inducing ligand (TRAIL); total cholesterol (TC); transcription factor Jun (c-Jun); transcription factor Nuclear factor of activated T cells c1 (NFATc1); Fos proto-oncogene (c-Fos); transforming growth factor β1 (TGF-β1); transmembrane protease serine 2 (TMPRSS2); triglycerides (TG); tryptophan hydroxylase-1 (TPH-1); tumor necrosis factor receptor associated factor 6 (TRAF6); tumor necrosis factor-α (TNF-α); tyrosine hydroxylase (TH); ultraviolet B (UVB); uncoupling protein 2 (UCP-2); urinary albumin excretion rate (UAER); urinary albumin-to-creatinine ratio (UACR); vacuolar-type ATPase (V-ATPase); vascular cellular adhesion molecule-1 (VCAM-1); vascular endothelial growth factor (VEGF); vascular endothelial growth factor receptor 2 (VEGFR2); zonula occludens-1 (ZO-1); α-smooth muscle actin (SMA-α).
